# Importance of Post-Translational Modifications for Functionality of a Chloroplast-Localized Carbonic Anhydrase (CAH1) in *Arabidopsis thaliana*

**DOI:** 10.1371/journal.pone.0021021

**Published:** 2011-06-10

**Authors:** Stefan Burén, Cristina Ortega-Villasante, Amaya Blanco-Rivero, Andrea Martínez-Bernardini, Tatiana Shutova, Dmitriy Shevela, Johannes Messinger, Laszlo Bako, Arsenio Villarejo, Göran Samuelsson

**Affiliations:** 1 Department of Plant Physiology, Umeå Plant Science Centre, Umeå University, Umeå, Sweden; 2 Department of Biology, Universidad Autónoma de Madrid, Madrid, Spain; 3 Department of Chemistry, Umeå University, Umeå, Sweden; Iowa State University, United States of America

## Abstract

**Background:**

The *Arabidopsis* CAH1 alpha-type carbonic anhydrase is one of the few plant proteins known to be targeted to the chloroplast through the secretory pathway. CAH1 is post-translationally modified at several residues by the attachment of N-glycans, resulting in a mature protein harbouring complex-type glycans. The reason of why trafficking through this non-canonical pathway is beneficial for certain chloroplast resident proteins is not yet known. Therefore, to elucidate the significance of glycosylation in trafficking and the effect of glycosylation on the stability and function of the protein, epitope-labelled wild type and mutated versions of CAH1 were expressed in plant cells.

**Methodology/Principal Findings:**

Transient expression of mutant CAH1 with disrupted glycosylation sites showed that the protein harbours four, or in certain cases five, N-glycans. While the wild type protein trafficked through the secretory pathway to the chloroplast, the non-glycosylated protein formed aggregates and associated with the ER chaperone BiP, indicating that glycosylation of CAH1 facilitates folding and ER-export. Using cysteine mutants we also assessed the role of disulphide bridge formation in the folding and stability of CAH1. We found that a disulphide bridge between cysteines at positions 27 and 191 in the mature protein was required for correct folding of the protein. Using a mass spectrometric approach we were able to measure the enzymatic activity of CAH1 protein. Under circumstances where protein N-glycosylation is blocked *in vivo*, the activity of CAH1 is completely inhibited.

**Conclusions/Significance:**

We show for the first time the importance of post-translational modifications such as N-glycosylation and intramolecular disulphide bridge formation in folding and trafficking of a protein from the secretory pathway to the chloroplast in higher plants. Requirements for these post-translational modifications for a fully functional native protein explain the need for an alternative route to the chloroplast.

## Introduction

Carbonic anhydrase (CA) is a ubiquitous zinc metalloenzyme that catalyzes the reversible interconversion of carbon dioxide (CO_2_) and bicarbonate (HCO_3_^−^) at very high turnover rates [1]. CA is required in biological systems since the uncatalyzed interconversion between these molecules is too slow to maintain the flux rates that may be required in living cells. Reactions catalyzed by CA may influence carboxylase and decarboxylase rates. Therefore, processes such as ion regulation, ion exchange and inorganic carbon supply, which are essential for efficient photosynthesis and respiration, are also influenced by the enzyme [2], [3]. The known CAs can be grouped into four families, called α-CA, β-CA, γ-CA and δ-CA [4], [5], [6]. Interestingly, these four families have no primary sequence similarities and hence they represent an important example of convergent evolution of catalytic activity. Plant CAs belong to α, β and γ-classes of CAs [2].

The *Arabidopsis thaliana* genome contains at least eight genes encoding α-type CAs (AtαCA1-8). CAH1 (AtαCA1) is an α-type CA in *Arabidopsis* and contains all 15 conserved catalytic and zinc-binding residues typical for active α-CAs [7]. SignalP [8] predicts that it contains an N-terminal signal peptide that directs the protein to the endoplasmic reticulum (ER) [9], where the polypeptide is N-glycosylated before being further targeted to the *Arabidopsis* chloroplast [7]. To date, only three other glycoproteins localized in the chloroplast of higher plants have been described: α-amylase I-1 [10], [11], α-amylase 3 [12], [13] and nucleotide pyrophosphatase/phosphodiesterase 1 [14], all three from rice. However, to our knowledge CAH1 is the only N-glycosylated protein identified experimentally in the chloroplast proteome of *Arabidopsis*, making it an interesting model protein from a general cell biological perspective. The majority of chloroplast proteins are encoded by the nuclear genome and cross the double membrane chloroplast envelope through the well-known Toc/Tic import complex in an unfolded state [15]. The advantages conferred by trafficking a few proteins through the secretory pathway before entering the chloroplast, are still unknown. However, since one of the main functions of protein N-glycosylation is believed to be related to protein folding [16], [17], we hypothesized that trafficking through this alternative pathway might be dictated by the need for post-translational modifications, such as N-glycosylation, for proper folding, and to enhance stability and/or function of these proteins.

In order to study the importance of endomembrane-specific post-translational modifications for correct trafficking and functioning of a chloroplast-localized protein, an epitope-tagged version of CAH1 was constructed that enabled both stable and transient expression in *Arabidopsis* suspension culture cells and protoplasts. In addition, several point-mutated versions were cloned and transiently expressed in protoplasts to explore the characteristics and function of the carbohydrate structures anchored to the protein and the importance of a putative intramolecular disulphide bridge in the protein.

In this paper we present the first thorough biochemical analysis of a protein trafficked through the secretory pathway to the higher plant chloroplast. We show that CAH1 is glycosylated at four, and in some cases five, sites and that glycosylation is necessary for correct folding, trafficking and functionality of the protein. Conversely, the non-glycosylated protein formed aggregates and was retained in the ER, associated with ER chaperones, indicating that glycosylation of CAH1 facilitates folding and ER-export. In addition, we demonstrate that the *Arabidopsis* CAH1 contains an intramolecular disulphide bridge between Cys27 and Cys191 in the mature protein, the presence of which is required for correct folding of the protein. Using a mass spectrometric method we showed that CAH1 is an active carbonic anhydrase isoform and that N-glycosylation is required for production of an active CAH1. The results provide valuable first indications of the reasons why some proteins are trafficked to the chloroplast through the secretory system.

## Results

### Expression of HA-tagged CAH1 results in a highly heterogeneous glycoform pattern

**Figure 1:**
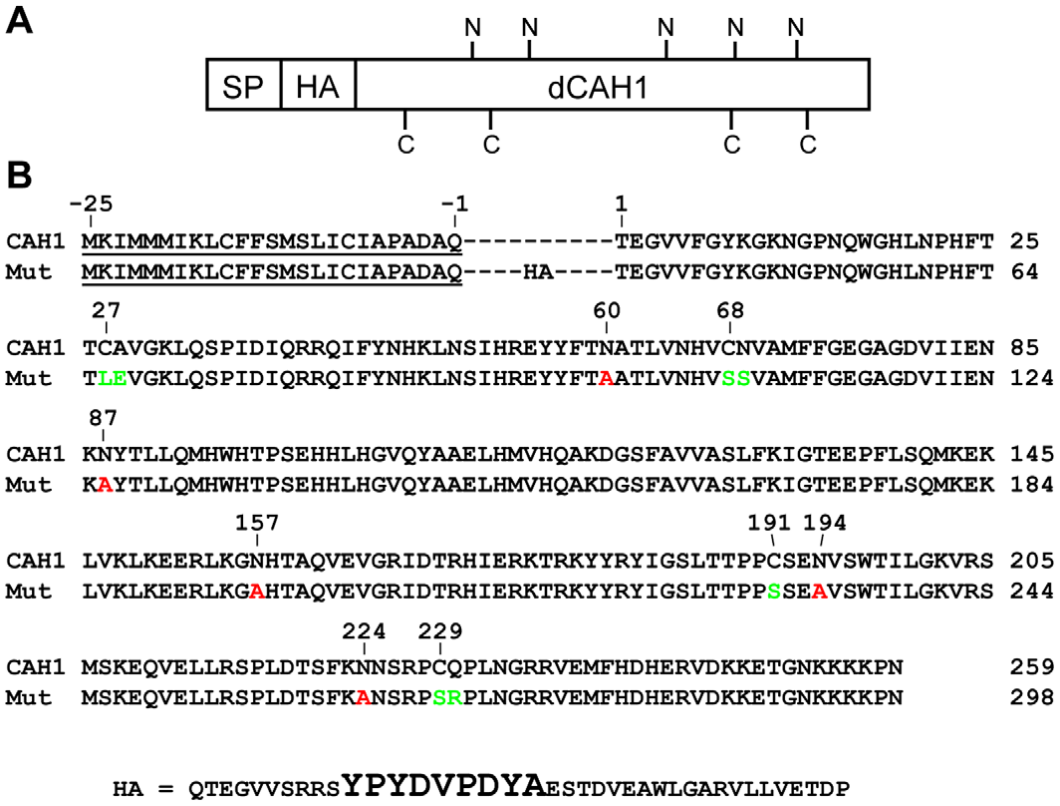
Schematic view and amino acid sequence of the HA epitope-tagged CAH1 variants. (A) The five potential N-glycosylation sites and the four cysteine residues are marked by “N” and “C”, respectively, and numbered according to their position in the mature native CAH1. SP, ER signal peptide; HA, HA-epitope tag; dCAH1, mature CAH1. (B) Position and sequence of the HA-tag is indicated together with the mutated cysteine residues (green) and N-glycosylation sites (red). HA epitope sequence is enlarged.

**Figure 2:**
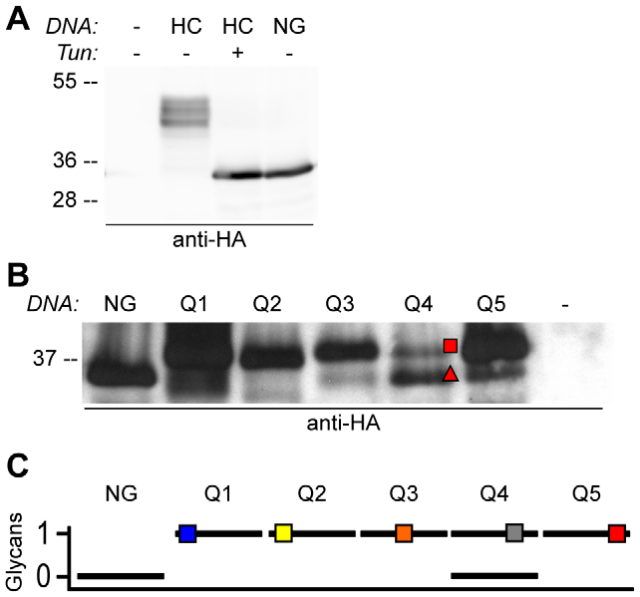
HA-tagged CAH1 is glycosylated in plant cells, harbouring four or five N-glycans. Migration patterns of HA-tagged CAH1 forms in protoplasts of *Arabidopsis* suspension culture cells (A), and *Arabidopsis* mesophyll cells (B), expressing wt (HC) or mutant CAH1 (NG, Q1–Q5). Antibody specificity was verified using water-transfected protoplasts (−). (A) Difference in protein migration due to glycosylation of HC, as compared to non-glycosylated tunicamycin (Tun)-treated HC and non-glycosylated mutant protein (NG). (B) Removal of four out of five potential N-glycosylation sites (Q1–Q5) results in polypeptides that migrate slower than the NG protein. Q4 mainly migrates as the NG mutant (triangle), but also as single glycosylated polypeptide (square). A band corresponding to non-glycosylated protein can also be seen for the Q1, Q3 and Q5 mutants, although to a much lesser extent than for the Q4 mutant protein. (C) Schematic view of the migration pattern in (B). Squares show the attachment of single N-linked glycans in the different glycoforms. Blue square represents NAT60; yellow square represents NYT87; orange square represents NHT157; gray square represents NVS194; red square represents NNS224.

CAH1 was previously identified as an α-type CA localized in the chloroplast of *Arabidopsis* [7]. The mature protein has five potential N-glycosylation sites and contains four cysteine residues. To study the function of these groups several mutant variants of the protein were generated, based on a Hemagglutinin (HA) epitope-tagged version of CAH1 (Figure 1, Table 1, Table S1). The HA-tagged CAH1 (HC) was stably transformed into an *Arabidopsis thaliana* cell suspension culture and sub-cellular localization of the expressed protein was analyzed using immunogold (IG) labelling followed by electron microscopy. The wild type HC was mainly localized to the chloroplast of cells, where the highest immunogold labelling density was observed (Table 2, Figure S1). As evident from Table 2, some labelling associated with the ER and Golgi apparatus was also detected. However, comparison of the labelling densities over the chloroplast and the secretory pathway compartments clearly indicated that the vast majority of the HC molecules were localized to the plastids in these cells, as previously shown for the native CAH1 [7]. In addition, like the native leaf protein, the HC protein was glycosylated (Figure 2a). Transient expression of HC in the presence of the glycosylation inhibitor tunicamycin (tun) resulted in a protein that migrated with the same apparent molecular weight as the non-glycosylated NG mutant protein, while HC protein from protoplasts not exposed to tunicamycin resulted in a complex pattern of higher molecular weight polypeptides indicating accumulation of distinct glycoforms of HC (Figure 2a), according to the mass difference previously observed for deglycosylated native protein *in planta* [7].

**Table 1:** Nomenclature of constructs used to transfect plant cells and protoplasts.

Clone	Point mutation	Mutated sites	Description
HC	-	-	HA-tagged wt CAH1
C1	CA27LE	Cysteine 1	
C2	CN68SS	Cysteine 2	
C3	C191S	Cysteine 3	
C4	CQ229SR	Cysteine 4	
C1+C3		Cysteine 1 and 3	= C1+C3
N1	NAT60AAT	Glycosylation site 1	
N2	NYT87AYT	Glycosylation site 2	
N3	NHT157AHT	Glycosylation site 3	
N4	NVS194AVS	Glycosylation site 4	
N5	NNS224ANS	Glycosylation site 5	
Q1		Glycosylation site 2, 3, 4 and 5	= N2+N3+N4+N5
Q2		Glycosylation site 1, 3, 4 and 5	= N1+N3+N4+N5
Q3		Glycosylation site 1, 2, 4 and 5	= N1+N2+N4+N5
Q4		Glycosylation site 1, 2, 3 and 5	= N1+N2+N3+N5
Q5		Glycosylation site 1, 2, 3 and 4	= N1+N2+N3+N4
NG		All glycosylation sites	= N1+N2+N3+N4+N5

**Table 2:** Intracellular distribution of HC protein in *Arabidopsis* suspension cultured cells stably expressing HA-tagged CAH1.

Subcellular compartment	Immunogold density (gold particles µm-2)	
	WT	HC
Chloroplast	2.27±0.90	16.05±2.30*
ER	1.90±0.10	7.40±0.50*
Golgi	3.50±0.40	10.2±0.30*
Nucleus	12.2±0.90	11.0±0.20
Vacuole	0.50±0.01	0.9±0.05

Immunogold labelling density over several subcellular compartments was estimated in both, non-transformed (WT) and HC stably expressing cultured cells using HA antibodies (mean ± SD, n = 10 cellular sections, * = p<0.05).

The glycosylated polypeptides migrated as a set of four rather diffuse bands, indicating the presence of several glycoforms of the HC protein. To clarify whether these HA-tagged CAH1 glycoforms arose from the attachment of different types of N-glycans or different numbers of glycans, five quadruple mutants, each expressing a protein with four of the five potential N-glycosylation sites mutated to alanine, were generated (Q1–Q5; Table 1). When the four quadruple mutants in which only site 1, 2, 3 or 5 could be glycosylated (Q1, Q2, Q3 and Q5 respectively) were separated by SDS-PAGE, most polypeptides exhibited a higher molecular mass than the NG mutant protein. The altered migration was consistent with the size expected if only one N-linked glycan was attached (Figure 2b and c). By contrast, the Q4 mutant migrated as two discrete bands, one at the same size as the NG mutant and one at the same size as the other quadruple mutants, suggesting that the NVS194 site mutated in Q4 is only partly occupied by glycans. These results indicated that in the total pool of HC, NVS194 is only glycosylated in a fraction of the HC molecules while the other four glycosylation sites are mainly occupied by glycans, explaining some of the glycoforms seen in Figure 2a.

To confirm that HC generally harbours four or five N-linked glycans, additional constructs with mutated glycosylation sites were tested, enabling analysis of HA-tagged CAH1 with one, two, three, four or five glycosylation sites removed (Table 1, Table S1). Elimination of each site shifted the migration pattern, creating a ladder of glycoforms ranging from fully glycosylated to non-glycosylated, confirming that all five sites in the HC protein can harbour N-linked glycans (Figure S2a). As previously observed, proteins carrying mutated glycosylation site NVS194 (N4, N3+N4, N4+N5, N3+N4+N5, Q1, Q2, Q3 and Q5) migrated in a different way compared to glycoforms in which this site remained intact (HC, N1, N2, N3, N5, N1+N2, N3+N5 and Q4). This observation was further confirmed by analysis of single mutant isoforms of HC (N1–N5; Table 1). Each mutation resulted in a protein with a slightly faster migration than the wild type HC protein (Figure S2b). In addition, mutant lacking glycosylation site NVS194 (N4) showed a different migration pattern compared to the other four single mutants, resulting in a simpler pattern of protein bands. Instead of changing the entire migration pattern of all the glycoforms, the forms of HC with the highest molecular masses appeared to be absent in cells expressing the N4 mutant (Figure S2c). These findings corroborated the hypothesis that HC is only partially glycosylated at NVS194, while the other four sites showed a more even glycosylation, explaining its complex migration pattern.

### HA-tagged CAH1 harbours different types of N-glycans

**Figure 3:**
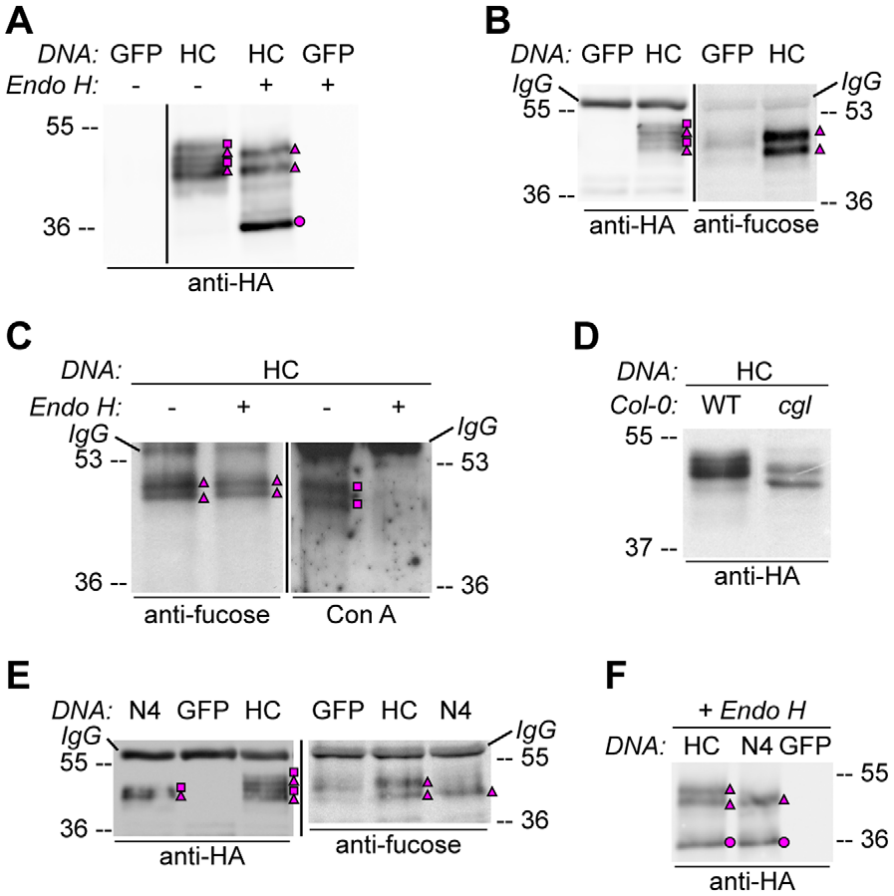
HA-tagged CAH1 contains both high mannose-type and complex-type N-glycans. Analysis of protein extracts of protoplasts from *Arabidopsis* suspension culture cells transiently expressing GFP (negative control), HA-tagged wt (HC) or single mutated (N4) CAH1. Heavy chain from immunoprecipitation is indicated as IgG. (A) HC protein migrates as four distinct bands: two isoforms are sensitive (squares) and two resistant (triangles) to Endo H. Deglycosylated protein is labelled with a dot. (B) HA-immunoprecipitation of HC reveals four protein isoforms when probed with HA antibodies, (squares and triangles) two of which are recognized by α(1,3)-fucose antibodies (triangles). (C) Endo H treatment of HC shows that the fucose containing glycoforms are resistant to the enzyme (triangles). The glycoforms detected by Con A affinoblot (squares) are sensitive to Endo H, corresponding to high mannose-type glycoforms. (D) HC migrates as two main bands when expressed in Columbia (Col)-0 mesophyll protoplasts from the *cgl* mutant, which is unable to produce complex-type N-glycans. (E) HA immunoprecipitation of HC reveals four protein isoforms, while only two can be detected of the N4 mutant using HA antibodies (triangles and squares). Analysis using α(1,3)-fucose antibodies on the same samples showed that only two of the HC, and one of the N4, glycoforms harbour complex-type glycans (triangles). (F) Endo H treatment and HA immunodetection of total protein extract confirmed that only one glycoform in N4 and two in HC are resistant to the enzyme (triangles). Deglycosylated isoforms are marked with dots.

When HC was separated on a gel with a lower acrylamide percentage that offered increased resolution of the different glycoforms, up to four bands could be visualized (Figure 3a). Protoplasts expressing GFP protein as a negative control were used to verify that all polypeptides detected by the HA antibodies resulted from expression of HA-tagged CAH1. As concluded from the data presented in Figure 2 and Figure S2a, N-glycosylation site NVS194 seemed to be partially occupied by glycans, resulting in two main glycoforms consisting of protein with either four or five glycans N-anchored to the polypeptide. Since the migration patterns indicated that cells expressing native HC produce at least four glycoforms of the protein (Figure 3a), it was important to elucidate the nature of these glycoforms. Therefore, HC protein extracts were treated with Endoglycosidase H (Endo H), prior to SDS-PAGE and Western blot analysis using HA antibodies. Endo H is an enzyme that deglycosylates glycoproteins harbouring high mannose type N-glycans typical of ER-localized glycoproteins [18], but not glycoproteins containing complex-type N-linked glycans that have been modified in the Golgi apparatus [19], [20], [21]. Analysis of migration patterns following deglycosylation using Endo H indicated that two of the four forms of HC with different masses are resistant to the enzyme, and thus likely to contain complex-type N-glycans. The other two glycoforms were readily deglycosylated, indicating that those glycoforms harbour high mannose-type N-glycans (Figure 3a).

To further confirm the presence of complex type N-linked glycans in Endo H-resistant glycoforms of HC, the proteins were immunoprecipitated using HA-agarose beads and immunodetected using fucose antibodies. These antibodies cross react to glycoproteins harbouring N-glycans that have been modified by α(1,3)-fucosyltransferase, a glycosyl transferase present in the Golgi apparatus of plants [22]. In agreement with the results of the deglycosylation experiment, the glycoforms apparently corresponding to the Endo H-resistant HC were detected by the fucose antibodies (Figure 3b). To address this possibility, Endo H treated HC protein was probed with anti-fucose antibodies (Figure 3c). This experiment confirmed that the Endo H resistant bands were also detected by anti-fucose, in addition to anti-HA, validating that two of the HC glycoforms are decorated with complex type N-glycans and that the protein is trafficked via the Golgi apparatus *en route* to the chloroplast (Figure 3b and c).Nature of the different glycoforms observed in HC was further assessed using the affinoblot technique. Concanavalin A (Con A) is a lectin that specifically binds to high mannose-type N-glycans. Since horse radish peroxidise also harbours this type of N-glycans, Con A can be detected by standard chemiluminiscence techniques [23]. Affinoblot assay showed that only two of the four bands observed in HC (see Figure 3a) were high mannose-type glycoforms (Figure 3c, right), and that following Endo H treatment, Con A failed to detect the deglycosylated bands observed in Figure 3a (dot), since no high mannose type glycans remain attached to the protein. An independent line of evidence suggesting that the HC pool is a mixture of complex- and high mannose-type glycoforms was obtained by expressing HC in protoplasts isolated from the *Arabidopsis cgl1* mutant. This mutant lacks a functional N-acetylglucosaminyltransferase I (GnT I), an enzyme required for N-glycans modification in the Golgi apparatus, and thus this mutant lacks the ability to synthesize complex-type N-glycans, and accumulates high-mannose type glycoproteins [24]. When HC was expressed in the *cgl1* background, the resulting protein migrated as two main bands, while several bands were detected from Col-0 protoplasts (Figure 3d). According to previous observations, two HC glycoforms corresponded to fucosylated glycans (Figure 3b). In order further analyse the nature of the glycans attached to HC, HA immunoprecipitated protein extracts of N4 single mutant were analysed. This mutant shows a simpler band pattern compared to HC, and when probed with fucose antibodies, only one glycoform can be detected (Figure 3e, pink triangles). Furthermore, Endo H treatment of N4 revealed that, according to fucose analysis (Figure 3e), only the lower-mass glycoform was resistant to deglycosylation (Figure 3f, pink triangle). This confirmed that mutation of glycosylation site four reduced the apparent number of glycoforms detected by SDS-PAGE and Western blotting to two, one with high mannose-type glycans and one with complex-type glycans.

**Figure 4:**
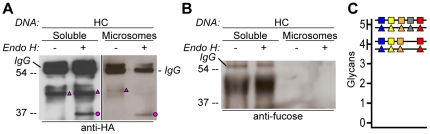
Glycosylation state of CAH1 found in the chloroplast stroma containing fraction is different than for microsome associated CAH1. Analysis of soluble (chloroplast stroma-containing) and microsome fractions of an *Arabidopsis* cell suspension culture stably expressing HC protein. Heavy chain from immunoprecipitation is indicated as IgG. (A) HA-immunoprecipitated HC from the soluble fraction is mainly resistant to Endo H (triangle), while most of HC from the microsome fraction was sensitive to Endo H (dot). (B) Only HA-immunoprecipitated protein from the soluble fraction can be detected using α(1,3)-fucose antibodies. (C) Schematic visualization of the proposed glycoforms of epitope-tagged CAH1 (blue, yellow, orange, grey and red squares and triangles symbolize N-glycosylation site 1–5, respectively). High-mannose type glycans are showed as squares and complex-type glycans as triangles.

As seen in the immunogold labelling experiment, some HC molecules were found outside the chloroplast in membrane-bound, presumably ER, structures (Table 2). To test whether Endo H susceptibility and resistance corresponded to different localizations of the expressed protein, soluble fractions (containing the soluble chloroplast stroma) and microsome fractions (enriched in ER, Figure S3) isolated from *Arabidopsis* suspension culture cells stably expressing HC were treated with Endo H. In accordance with expectations, the microsome fraction contained Endo H-sensitive HC, while the protein present in the soluble fraction was mainly Endo H-resistant (Figure 4a). In addition, only HC immunoprecipitated from the soluble fraction was detected by fucose antibodies (Figure 4b), confirming that the complex-type HC glycoforms were present in the soluble fraction while high mannose-type HC glycoforms were present in the microsome fraction, presumably corresponding to newly synthesized protein destined for the chloroplast.

In conclusion, the different glycoforms of the HC protein seem to differ in both number (mainly four or five) and type (high mannose or complex) of attached N-linked glycans, resulting in four distinct glycoforms (Figure 4c).

### Glycosylation of CAH1 is necessary for correct folding of the protein

**Figure 5:**
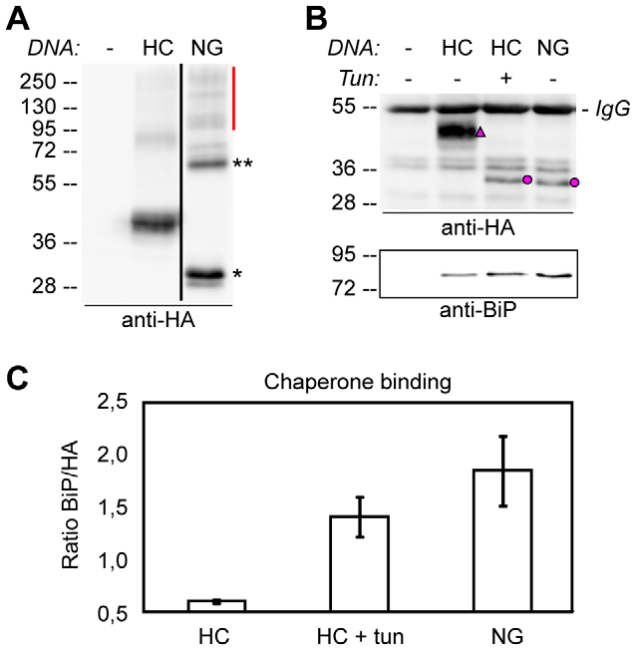
N-glycosylation is required for folding of CAH1. Protein extracts were prepared from *Arabidopsis* cell suspension culture protoplasts expressing either HA-tagged wt CAH1 (HC), in presence or absence of tunicamycin (Tun), or the non-glycosylated mutant version (NG). As negative controls, water-transfected protoplasts (−) were used. (A) Migration pattern of both HC and NG under non-reducing conditions. The presence of a potential dimer (**) of the monomeric NG protein (*) is indicated. In addition, high molecular weight aggregates (red dash) could be observed. Black line indicates different parts of the same membrane and exposure. (B) Analysis of the amount of BiP co-precipitated with HA-immunoprecipitated HC and NG (triangle and dots) using HA antibodies (upper panel) and BiP antibodies (lower panel). Heavy chain from immunoprecipitation is indicated as IgG. (C) Densitometric analysis of immunoblots shown in panel B in order to estimate relative amount of BiP co-precipitating with HC, HC treated with Tun (HC+tun), and NG mutant isoform (NG). The graph represents the ratio of BiP to HA-tagged protein (marked by triangle and dots) (mean ± SE, n = 2).

Glycosylation is known to affect protein folding and stability [9], [16], [17], [25], [26]. Therefore, compromised glycosylation might result retention of the misfolded protein in ER, followed by translocation out to the cytosol and degradation by the proteasome [9], [17], [27]. Under such conditions, non-native disulphide bridges can be formed, causing oligomerization and aggregation of the protein [16], [26]. To further study the effects of glycosylation on the HA-tagged CAH1 protein, HC and NG were analyzed under both reducing and non-reducing conditions. When non-glycosylated HA-tagged CAH1 protein (NG) was separated by SDS-PAGE under non-reducing conditions, a strong band of ca. 65 kDa was detected in addition to the monomeric protein (theoretical size 34 kDa), presumably corresponding to a dimer of the NG mutant protein (Figure 5a). A weak band possibly corresponding to a dimer of the glycosylated HC was also detected, but less abundant than the non-glycosylated NG, suggesting that glycosylation is important for correct folding and prevents formation of aggregates of CAH1.

Misfolded proteins are retained by the quality-control system in the ER (ERQC), which stabilizes and assists proper folding of proteins with the help of chaperones [28], [29]. One of the most thoroughly characterized ER-resident chaperones is the binding protein BiP, a member of the Hsc70 family of molecular chaperones [9], [30], [31]. During the quality control process, BiP attaches to misfolded proteins. Such interactions can be detected by immunoprecipitation of the misfolded protein, resulting in co-precipitation of BiP [32], [33]. If mutagenesis of glycosylation sites affected folding of HC, we postulated that immunoprecipitation of such misfolded polypeptides would co-precipitate BiP. To test whether such interactions could be detected, non-glycosylated HA-tagged CAH1 was immunoprecipitated using anti-HA agarose. Although the level of HA-tagged CAH1 was lower in the precipitate of the NG mutant protein (Figure 5b, upper panel), the relative amount of co-precipitated BiP was clearly higher than that precipitated with the glycosylated wt HC protein (Figure 5b, lower panel and Figure 5c). To confirm that this effect was not due to misfolding because of the mutations *per se*, HC was expressed in the presence of the glycosylation inhibitor tunicamycin, again resulting in increased amounts of co-precipitated BiP (Figure 5c). In conclusion, the results show that glycosylation is necessary for correct folding of the HC protein.

### Intramolecular disulphide bridges are important for folding and ER-export of the CAH1 protein

**Figure 6:**
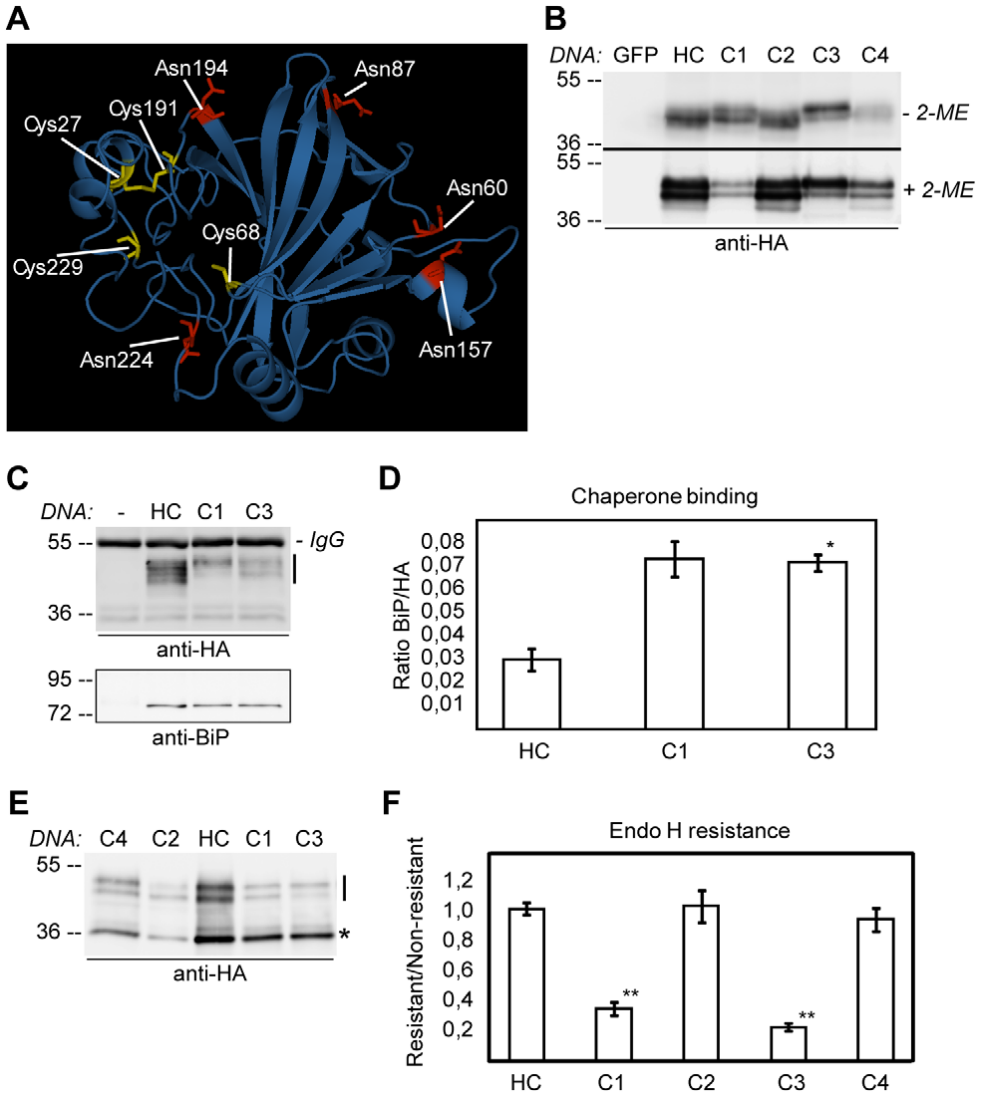
Intramolecular disulphide bridge in HA-tagged CAH1 is required for correct folding and N-glycan maturation. (A) Prediction of the 3D structure of CAH1 using SWISS-MODEL [48], [49], [50] and visualization using Swiss-PdbViewer [52] shows the five potential N-glycosylation sites (red) and the four cysteine residues of the mature CAH1 protein (yellow). (B) Immunoblot analysis with HA antibodies of protein extracts from *Arabidopsis* cell suspension culture transiently transfected with HA-tagged wt CAH1 (HC) and four cysteine mutant clones (C1–C4) separated under non-reducing (−2-ME) and reducing (+2-ME) conditions. As negative controls, protoplasts expressing GFP were used (GFP). (C) Protein extracts from GFP, HC, C1 and C3 transfected protoplasts were immunoprecipitated using HA-agarose and the level of co-precipitated BiP was analyzed by immunoblot with HA (upper panel) and BiP antibodies (lower panel). Immunoprecipitated CAH1 variants are marked by a line. Heavy chain from immunoprecipitation is indicated as IgG. (D) Densitometric analysis of immunoblots as shown in (D) to estimate the relative amount of BiP co-precipitating with HC, and mutant forms C1 and C3. The graph represents the ratio of BiP to HA-tagged protein (mean ± SE, * = p<0.05, n = 2). (E) Endo H-treatment of HC and cysteine mutant proteins. Endo H-resistant glycoforms are marked by a line, Endo H-sensitive glycoforms by a star. (F) Densitometric analysis of the relative amounts of Endo H resistant and sensitive glycoforms. The graph represents the ratio of Endo H resistant/sensitive glycoprotein (mean ± SE, ** = p<0.01, n = 4).

Mature CAH1 has four cysteines potentially involved in the formation of intra and/or intermolecular disulphide bridges (Figure 1). The modelled 3D structure of CAH1 protein indicates that Cys27 and Cys191 are located in close proximity to each other (Figure 6a). These cysteine residues are also conserved in α-CA homologues of *Arabidopsis* described by Fabre *et al.* [4] (Figure S4a).

To identify cysteine residues involved in intramolecular bond formation, each of the four cysteine residues were individually mutated (C1–C4; Table 1, Figure 1). Under reducing conditions, each mutant protein migrated with a similar pattern to wt HC (Figure 6b, lower panel). Under non-reducing conditions, C2 and C4 mutants showed similar migration patterns to HC, while C1 and C3 displayed similar migration patterns to each other, but apparently different from that of wt HC (Figure 6b, upper panel). In addition, the electrophoretic mobility of the C1+C3 double mutant was completely insensitive to the addition of reducing agents compared to the HC protein (Figure S4b and c).These findings are consistent with Cys27 and Cys191 (mutated sites in C1 and C3 respectively) forming an intramolecular disulphide bond, as predicted from the 3D model structure of the protein.

While HC, C1, C2 and C4 in the presence of 2-ME migrated as a double band in which each isoform was detected with comparable intensity, the C3 mutant protein was mainly present as the higher mass isoform (Figure 6b). As concluded in Figure 2 and Figure S2, incomplete glycosylation of HC, specifically from partial occupation of site NVS194, results in two major glycoforms of the protein. Since only two residues separate Cys191 from NVS194 (Figure 1b), and because Cys27 and Cys191 appear to form a disulphide bridge, the C3 mutation could promote accumulation of the protein isoform harbouring five glycans by making NVS194 more accessible to the glycosylation machinery. This could explain the prevalence of the higher mass isoform of C3 seen in Figure 6b.

Cys27 and Cys191 seem to be conserved in the α-type class of CAs in *Arabidopsis* (Figure S4a), and previous studies on human α-CA have shown the importance of intramolecular disulphide bridge for correct folding and activity of the protein [34]. Accordingly, we consistently detected lower levels of C1 and C3 mutant proteins than wt HC protein, indicating that this disulphide bridge is critical for folding and/or stability of the protein (data not shown). To test whether BiP showed increased affinity to these cysteine mutants, wt HC, C1 and C3 mutant proteins were immunoprecipitated, and the amount of co-precipitated BiP was analyzed. Despite the lower levels of precipitated C1 and C3 mutant proteins, the amounts of BiP that co-precipitated with them were similar to wt HC, indicating that folding of C1 and C3 is compromised, as seen from quantification of the relative amount of BiP that is binding to these mutant proteins (Figure 6c and d). In addition, Endo H treatment also showed that the C1 and C3 mutant polypeptides were significantly more susceptible to Endo H treatment, while no effect was observed when the other two cysteine residues (Cys68 and Cys229, corresponding to C2 and C4) were mutated (Figure 6e and f). These data suggest that C1 and C3 mutant proteins were not exported from the ER, but rather retained as unfolded/partly folded complexes with chaperones.

### N-linked glycans are needed for active CAH1

**Figure 7:**
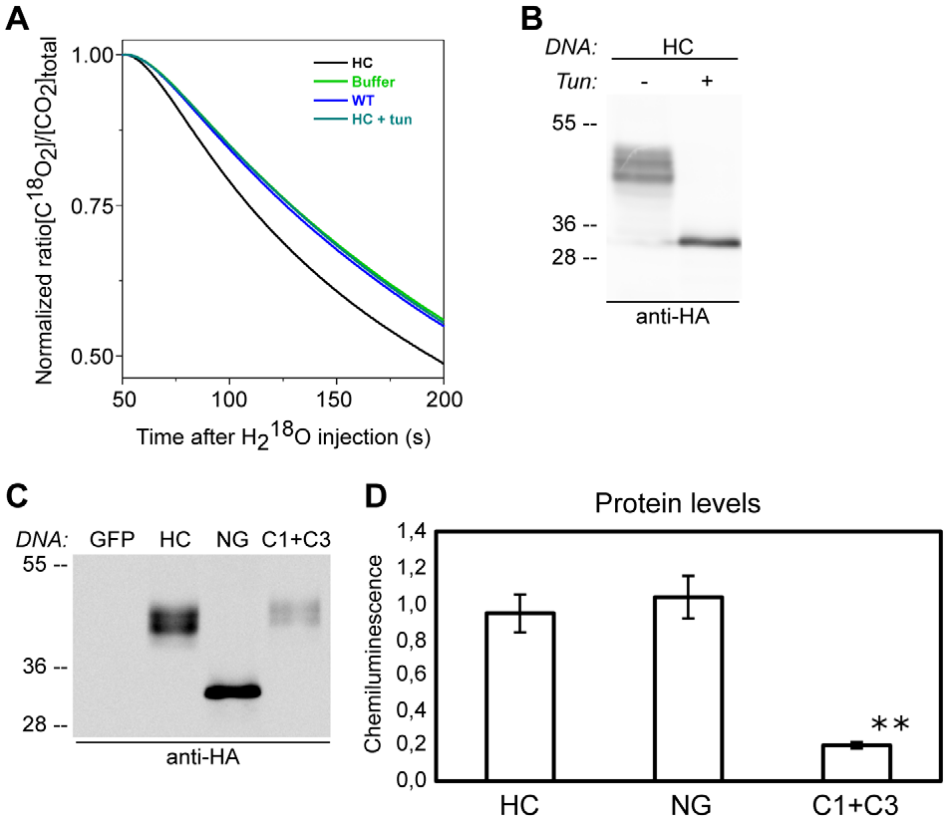
CA activity of the CAH1 protein requires N-glycosylation. (A) MIMS measurements of the change in C^18^O_2_ concentration as function of time after the 15-µl-injection and mixing of air-saturated H_2_^18^O (to final enrichment of 2.4%) into the 600 µl-MS cell at pH 6.5 and 20°C. Average traces of 3 repeats are presented, corresponding to HA-immunoprecipitates from cells stably expressing HA-tagged CAH1 (HC, black), HC treated with tunicamycin (cyano), buffer (green), or non-transformed cells (WT, blue). (B) Immunoblot analysis with HA antibodies of total extracts from *Arabidopsis* suspension cultured cells stably expressing HC, treated (+) and non-treated (−) with tunicamycin for 24 h. (C) Immunoblot analysis with HA antibodies of protein extracts from protoplasts over-expressing GFP (negative control), HC, non-glycosylated (NG) or double cysteine (C1+C3) mutant proteins. (D) Densitometric analysis of the protein levels in immunoblots as shown in (C) (mean ± SE, ** = p<0.01, n = 11 (C1+C3) or 12 (HC, NG)).

Our experimental data indicated that N-glycosylation and an intramolecular disulphide bridge formation are required for folding and thus ER export and trafficking of HC protein. In order to test the effects of these post-translational modifications on the activity of the CAH1 protein, we applied a strategy that allowed us to specifically measure the activity of CAH1 in the cellular background of numerous other CA isoforms. HC protein was immunoprecipitated from suspension culture cells stably expressing HC. The activity of the immunoprecipitated HC protein was then measured using a membrane-inlet mass spectrometer (MIMS) by monitoring the change in ^12^C^18^O^18^O concentration (*m/z* = 48) as a function over time after the injection of air-saturated H_2_^18^O (Table 3, Figure 7a). The activity was expressed as the rate constant (*k*, min^−1^) for mono exponential decay of the normalized ^12^C^18^O^18^O concentration. The activity of the immunoprecipitated HC protein was clearly detectable and the kinetics faster than a control containing buffer only. Samples prepared from culture cells not expressing HC showed activity values nearly identical to buffer control, indicating that our approach measures exclusively immunoprecipitated HC protein and not other CA isoforms (Table 3). Once the method was validated, the role of the N-linked glycans anchored to the HC protein was tested by performing the same experiment using immunoprecipitated HC protein from culture cells treated with tunicamycin for 24 h, a condition that mimics expression of the non-glycosylated mutant. This treatment completely blocked the N-glycosylation of HC, thus the only form that could be detected in these cells was the non-glycosylated one (Figure 7b). The non-glycosylated HC isoform did not show any detectable activity (Table 3). Western blot analysis showed that HC accumulated to comparable amounts in both control and tunicamycin treated cells (Figure 7b), indicating that the difference in activity was not due to differences in the amount of glycosylated and non-glycosylated isoforms.

**Table 3:** CA activity of CAH1 protein is affected by N-glycosylation.

Buffer	WT	HC	HC+Tun
0.20±0.02	0.21±0.01	0.30±0.02	0.19±0.01

Rate constants (*k*, min^−1^) for monoexponential decay of the normalized ^12^C^18^O^18^O concentration (Figure 7) after injection of air-saturated H_2_^18^O into H_2_^16^O buffer without (Buffer) or with HA immunoprecipitate from non-transformed culture cells (WT), or cells stably expressing HC protein in the absence (HC) or presence of tunicamycin (HC+Tun). Reactions were performed at 20°C, pH 6.5 (mean ± SE, n = 3).

The importance of the disulphide bridge for the activity of CAH1 was difficult to estimate. The level of the C1+C3 mutant protein, when transiently expressed in the protoplast system, was always much lower than the wt and the NG isoforms (Figure 7c and d). However, the presence of reducing agents has already been shown to inhibit the activity of alpha-type CAs [34], [35], [36] and this has been used as an argument to emphasize the relevance of the disulphide bridge formation for the activity of CAs in general.

## Discussion

*Arabidopsis* have three types of CAs; α, β, and γ-CA [2], distributed in various cellular locations such as the mitochondria, chloroplasts, cytosol and periplasmic space. Little is known about the specific roles of CAs in C3 photosynthesis. However, early work on the chloroplast β-type CA showed that this isoform does not play a crucial role in the photosynthetic processes of the plant [37]. In addition to the highly abundant chloroplast β-type CA, representing some 0.5–2% of total soluble leaf protein [38], *Arabidopsis* stroma contains an α-CA (CAH1). CAH1 is the only CA shown to be targeted to the chloroplast stroma through the endomembrane system [7], although most α-CA genes present in *Arabidopsis* [4] have predicted ER signal peptides (Figure S5). The occurrence of additional stroma CAs, such as CAH1, could now explain some of the early data on the physiological functions of chloroplast CA activities. Another important issue to be addressed is why CAH1 is trafficked through an alternative route to the chloroplast instead of being post-translationally targeted through the Toc/Tic complex like the vast majority of plastid proteins [15].

We have previously shown that CAH1 has several unexpected structural features for a chloroplast-localized protein [7]. In addition to the presence of an N-terminal targeting signal peptide directing the protein to the ER, the CAH1 polypeptide is N-glycosylated before it is targeted to the *Arabidopsis* chloroplast. To our knowledge, CAH1 is the only N-glycosylated chloroplast protein found in *Arabidopsis* so far. The machinery whereby this protein is targeted to the chloroplast is unknown, although it was recently shown that the rice α-amylase I-1 glycoprotein is trafficked to the plastid by membrane vesicles in an ARF1- and SAR1-dependent manner when expressed in onion epidermal cells [11].

To assess the biological significance of possession of a set of chloroplast glycoproteins targeted through the endomembrane system and explore the relationships between the structure, function and novel trafficking route to the chloroplast of CAH1, we expressed an epitope-tagged version of the wt protein (HC). Several mutant versions of the HC protein were also generated and transiently expressed in protoplasts to evaluate the role of the attached carbohydrate structures and its redox status (Figure 1, Table 1, Table S1).

Our data clearly showed that the epitope-tagged CAH1 protein was targeted to the chloroplast in *Arabidopsis* suspension culture cells. Additionally, immunogold labelling and electron microscopy localized the HC protein in cellular compartments *en route* to the chloroplast, e.g. the ER and Golgi apparatus (Table 2). This localization pattern was further supported by biochemical analyses of total cell protein extracts, showing the occurrence of glycoforms harbouring both high mannose-type N-glycans characteristic of ER-localized proteins (sensitive to EndoH), and complex-type N-glycans modified by glycosyltransferases and glycosidases present in the Golgi apparatus (resistant to EndoH) (Figure 3 and 4). Subcellular fractionation of suspension culture cells stably expressing the HC protein showed that the Endo H-resistant glycoform was present in the soluble, stroma-containing fraction, while the Endo H-sensitive HC glycoforms were present in the ER-enriched microsome fraction. These results indicated that the protein associated with the endomembrane system in the immunogold localization experiment presumably represents protein destined for the chloroplast, but not yet exported from the ER and therefore detected as Endo H-sensitive glycoprotein. Although different glycoforms of HC were observed (high mannose- and complex-type), we did not detect any non-glycosylated protein in our protein extracts, confirming that the N-terminal sequence of CAH1 is recognized as a signal peptide and co-translationally inserted into the ER lumen [7].

A possible explanation for the difference in subcellular localization between the over-expressed protein and the native CAH1 is that the strong 35S promoter may cause saturation of the targeting machinery, resulting in retention of some of the protein in the ER and Golgi apparatus, and hence accumulation of different glycoforms of the HA-tagged protein. In agreement with this hypothesis, over-expression of the rice α-amylase I-1 glycoprotein seems to activate Golgi-to-plastid vesicle trafficking in onion epidermal cells, indicating that trafficking of glycoproteins to the plastid in higher plants is tightly regulated [11]. Over-expression of HC might therefore result in higher levels of the chloroplast-targeted glycoprotein than the targeting machinery can cope with, leading to accumulation of glycoforms in the ER/Golgi system and hence delayed trafficking. Accordingly, we observed that cycloheximide (CHX) treatment reduced the abundance of HC molecules harbouring high mannose-type N-glycans, indicating that these isoforms represent newly synthesized proteins still trafficking through the secretory pathway *en route* to the chloroplast (Figure S6). These results confirmed the findings of Endo H analysis of HC protein in soluble and microsome fractions (Figure 4a and b), and presumably explain why proteins could be detected *en route* to their final location in the immunogold labelling experiment (Table 2).

Alternatively, the presence of Endo H-sensitive HC glycoforms might be due to misfolding of the over-expressed protein. Proteins unable to fold correctly in the ER are retained in this organelle by chaperone complexes and when refolding is unsuccessful misfolded proteins are degraded [9], [17], [29]. Addition of CHX arrests biosynthesis of HC proteins, and since new proteins cannot be synthesized the amount of misfolded proteins decrease. Degradation of the misfolded and high mannose-type ER localized proteins should result in an increase in the relative amount of correctly folded glycoproteins harbouring complex-type N-glycans that should theoretically be exported from the ER and targeted to the chloroplast, acquiring Endo H resistance on the way, and thus a higher Endo H-resistant/sensitive ratio, is detected (Figure S6). The amounts of BiP, an ER-resident chaperone involved in protein translocation and folding [31], [32], [33] that co- immunoprecipitated with HC (Figure 5b) also indicate that folding of HC is impaired or slow when the protein is over-expressed, resulting in increased ER localization and Endo H-sensitivity of the protein pool. This could also explain the presence of HC in the ER detected by immunogold labelling and electron microscopy (Table 2).

Our previous study of the CAH1 protein showed that the protein is N-glycosylated, but the numbers and positions of the glycans *in vivo* were not resolved [7], therefore mutants to study glycosylation status were prepared (Figure 1, Table 1, Table S1). The electrophoretic migration pattern of the NG mutant protein was identical to that of HC expressed in the presence of the N-glycosylation inhibitor tunicamycin (Figure 2a), confirming that no additional sites other than the five mutated could harbour N-glycans in the CAH1 protein. The analysis of the single and quadruple glycosylation mutants showed that while N-glycosylation sites NAT60, NYT87, NHT157and NNS224 mostly harboured N-linked glycans, site NVS194 was to a lesser extent occupied by an N-glycan (Figure 2b and c, Figures S2b and c). These findings are supported by *in vitro* uptake studies of CAH1 into dog pancreas microsomes, which resulted in translocated and signal peptide-processed protein. This studies showed that CAH1 migrated predominantly as two bands in an SDS-PAGE, indicating that regardless of the eukaryotic system used for expressing the protein, CAH1 consistently appears as two glycoforms differing in the number of N-linked glycans attached to the polypeptide [7].

As discussed above, immunogold labelling and electron microscopy indicated that the HC protein was not exclusively localized to the chloroplast since some of the protein was also detected in the ER. Analysis of Endo H-resistance/sensitivity in the transient expression system clearly showed that two of the four observed bands in HC harboured high mannose-type N-glycans, while the other two contained complex-type N-glycans (Figure 3). The presence of α(1,3)-fucose residues in these glycoforms confirmed that they had been exported from the ER to the Golgi apparatus, also corroborated by the fractionation analysis (Figure 4a and b).

In conclusion, expression of HC results in at least four glycoforms, two of which are due to partial glycosylation of NVS194, resulting in either four or five glycans attached to the protein. Each of these two glycoforms can in addition, harbour high mannose or complex type glycans, depending on the subcellular location of the protein. The reason for the partial glycosylation of site four is currently unknown. However, glycosylation site NVS194 is located close to Cys191, which forms a disulphide bond to Cys27 (Figure 6), and perhaps this bond cannot be created if NVS194 is glycosylated. Native CAH1 sometimes migrates as a double band [7], but whether the weak extra band is due to partial glycosylation of NVS194 is currently unknown.

Glycosylation of proteins is known to be important for folding and stability [16], [17]. Although most of our current knowledge in quality control and glycan processing in the ER comes from studies of yeast and mammalian systems, most components seem to be conserved in plants [17], [29]. Misfolded polypeptides are detected by the ER quality control system that ensures export of correctly folded proteins. Removal of two of the three terminal glucose units of the oligosaccharide precursor Glc_3_Man_9_GlcNAc_2_ by the action of glucosidases I and II in the ER [22], results in monoglucosylated glycans that are attracted to the lectin chaperones calnexin and calreticulin. This binding slows down the folding process and increases the overall efficiency of correct disulphide bonds formation [9]. Glucosidase II trims the last glucose unit before export from the ER [9], [22]. Misfolded glycoproteins are sensed by UDP-glucose∶glycoprotein glucosyltransferase, which re-glucosylates the protein [9], [39], resulting in monoglucosylated oligosaccharides that again are attracted to the lectin chaperones that assist in refolding of the protein [16], [29]. Glycoproteins that fail to fold correctly are generally found in the ER as large aggregates that are non-covalently bound to BiP and other ER chaperones, before eventually being translocated to the cytosol for degradation by the proteasome [9], [17], [28], [29]. The ER aggregates are often covalently linked to each other by non-native disulphide bonds [17]. These aggregates can be detected upon separation by SDS-PAGE under non-reducing conditions to keep disulphide bridges intact [26]. Our data show that a CAH1 isoform lacking all five N-glycosylation sites forms such aggregates when separated under non-reducing conditions. Not only a dimeric isoform, but also high molecular weight aggregates were observed under such conditions, suggesting the formation of non-native disulphide bonds between mutant monomers (Figure 5a). Impaired folding of the NG mutant was further confirmed by quantification of co-precipitated BiP. Both NG mutant protein and HC from cells treated with tunicamycin co-precipitated larger amounts of BiP compared to the glycosylated HC protein (Figure 5b and c). These data indicate that glycosylation of CAH1 is necessary for correct folding and that, in the absence of N-glycans attached to the polypeptide chain, HA-tagged CAH1 is associated with ER chaperones and likely retained in this compartment. The analysis of the migration patterns of the single and quadruple glycomutants further confirms this hypothesis. Several HC forms with different molecular masses were detected in extracts from systems expressing the single glycosylation mutants, corresponding to the different numbers and forms of N-linked glycans (Figure S2b), as discussed above. However, the quadruple mutants showed a simpler migration pattern (Figure 2b and c), presumably indicating that they each contain only one type of N-glycan (high mannose), and that one glycan is not sufficient for correct folding of the protein and trafficking from the ER to the Golgi. The higher resistance to Endo H of the single mutants compared to the quadruple mutants confirm our contention and suggests that one glycan is not sufficient for folding and ER-export (data not shown).Whether the protein is decorated with high mannose- or complex-type N-glycans does not appear to affect the final destination of the protein, since CAH1 is targeted to the chloroplast stroma in the *cgl1* mutant, which is unable to synthesize complex N-glycans (Ortega-Villasante C., Burén S., and Villarejo A., unpublished data).

Disulphide bridges have previously been shown to be required for correct folding and activity of the human α-type CA IV, in which an intramolecular disulphide bond between Cys28 and Cys211 is present in the native protein [34]. Mature CAH1 has four cysteine residues, two of which seem to be conserved among α-type CAs in *Arabidopsis* (corresponding to Cys27 and Cys191 in CAH1, Figure S4a). The data presented in this study strongly indicate that a disulphide bridge is formed between Cys27 and Cys191 in the mature native protein. This finding is consistent with the disulphide bridge found in human CA IV [34] and could be a result of the close proximity between Cys27 and Cys191 in the predicted 3D structure of CAH1 (Figure 5a). Our results indicate that the C1 and C3 mutant proteins mainly harbour high mannose-type glycans (Figure 6e and f), suggesting that the intramolecular disulphide bridge is important for folding, and that the protein cannot be exported from the ER when this disulphide bridge is absent. The C1 and C3 mutant proteins were not only retained in the ER but were also misfolded and bound to BiP (Figure 6c and d). Thus, as reported for the human CA IV [34], the formation of an intramolecular disulphide bridge between Cys27 and Cys191 is required for the stability and correct folding of the CAH1 protein.

Our results established that N-glycosylation and formation of an intramolecular disulphide bridge are crucial steps in CAH1 folding and indicated that these post-translational modifications are required for CAH1 to adopt a stable conformation. The absence of either N-glycan decoration, or the formation of the disulphide bond, leads to retention of CAH1 in the ER in an unfolded state. We have set up a highly sensitive mass spectrometric-based method that allows measuring of the specific activity of CAH1 without interference from other CA isoforms. The measurements show firstly, that CAH1 is an active plastid CA and moreover, that the absence of N-linked glycans anchored to the protein results in a completely inactive CAH1 isoform. The requirement of an intramolecular disulphide bridge for CA activity could not be determined in our experiments. However, it has been previously reported and it is described in the literature that the presence of reducing agents such as dithiothreitol inhibits the activity of α-type CAs [34], [35], [36] (Shutova and Samuelsson, unpublished data). These findings indicate that a disulphide bridge may also be crucial for the activity of CAH1. The results clearly show that N-glycosylation not only affected folding (and trafficking) of the protein, but also its ability to catalyze the reaction in which carbon dioxide is converted to bicarbonate.

In conclusion, we show for the first time the importance of N-glycosylation for a protein targeted to the chloroplast in higher plants. CAH1 is an α-type CA that contains several glycosylation sites that must be occupied by N-glycans for correct folding of the protein. Most other α-type CA proteins contain one or more potential glycosylation sites, suggesting that glycosylation might be a general structural element required for folding of this family of proteins. We have further shown that the protein needs to be stabilized by a disulphide bridge between the conserved Cys27 and Cys191 residues for correct folding and ER-export. Moreover, the enzymatic activity of HA-tagged CAH1 lacking N-linked glycans is severely compromised. Collectively, these results indicate that glycosylation and disulphide bond formation are key requirements for CAH1 protein folding and trafficking. Most importantly, glycosylation, which takes place exclusively in the endomembrane system, acts to maintain CAH1 protein structure and thus might explain the existence and preservation of the trafficking pathway to the chloroplast through the endomembrane system. Since the unfolding required for a protein to cross the chloroplast envelope through the Toc/Tic complex would disrupt its structure and function it is anticipated that uptake of CAH1 into the chloroplasts takes place via an alternative mechanism. It remains to be tested whether CAH1 trafficking involves fusion of membrane vesicles in a similar fashion to that recently described for rice α-amylase I-1 glycoprotein [11].

## Materials and Methods

### Primers used for cloning of HA-tagged CAH1 variants

for-pUC18 GTTTTCCCAGTCACGAC

rev-XbaI CTAGATCTAGACACTACTCCTTCTGTCT

for-BamHI CAAGAGGATCCTCAGACAGAAGGAGTAG

rev-BamHI TATGGATCCTTAATTGGGTTTTTTCTTT

ForCAH1/KpnI TTACAGGTACCATGAAGATTATGATGATGA

RevCAH1/SacI ACTTTGAGCTCAAATGTTTGAACGAGAATT

N1for TACTACTTCACAGCCGCAACACTAGTGAA

N1rev TTCACGGTGTATTGAATTCAATTTG

N2for ATAGAAAACAAGGCCTATACCTTACTGCAAAT

N2rev TATCACATCTCCTGCTCCCTCCC

N3for AGACTCAAAGGGGCCCACACAGCACAAG

N3rev CTCTTCCTTTAGCTTCACCAATTTCTC

N4for CCTTGCTCCGAGGCCGTTTCTTGGAC

N4rev AGGAGTAGTGAGTGAACCAATGTATCT

N5for ACTTCTTTCAAGGCCAATTCAAGACCGT

N5rev GTCCAATGGAGATCTGAGTAGTTCTAC

C1 for ACACTCGAGGTCGGTAAATTGCAATCTCCA

C1 rev GACCTCGAGTGTGGTGAAGTGAGGGTTTAA

C2 for CGTCTCGAGTGTTGCCATGTTCTTCGGGGA

C2 rev ACACTCGAGACGTGGTTCACTAGTGTTGCG

C3 for TCCCTCGAGCGAGAACGTTTCTTGGACCAT

C3 rev TCGCTCGAGGGAGGAGTAGTGAGTGAACCA

C4 for ACCCTCGAGACCCCTCAACGGCCGGAGAGT

C4 rev GGTCTCGAGGGTCTTGAATTGTTCTTGAAA

### Cloning of wt and mutant variants of HA-tagged CAH1

To create N-terminally HA epitope-tagged CAH1, the signal peptide of CAH1 under the control of a 35S promoter was amplified from CaMV35S-CAH1-sGFP(S65T) [7] by PCR with for-pUC18 and rev-XbaI primers. The PCR fragment was digested with BamHI, and end-blunted by Klenow filling, followed by XbaI digestion. The digested fragment was subcloned 5′ of the sequence coding for the HA epitope in pPE1000 NanoT [40], digested by XhoI and XbaI (following end-blunting of the XhoI site by Klenow filling), to replace the double 35S promoter with the single 35S promoter signal peptide sequence. The remaining CAH1 sequence was PCR-amplified from CaMV35S-CAH1-sGFP(S65T) with for-BamHI and rev-BamHI primers, digested and subcloned 3′ of the HA tagged CAH1 signal peptide of BamHI-digested pPE1000 NanoT plasmid, to create pSBHACAH1.

Mutated glycoforms of HA-tagged CAH1 were created by site-directed mutagenesis using a Phusion Site-Directed Mutagenesis Kit (FINNZYMES OY, Espoo, Finland). Single mutations of pSBHACAH1 (N1, N2, N3, N4 and N5) were created using the 5′-phosphorylated primer pairs N1for and N1rev, N2for and N2rev, N3for and N3rev, N4for and N4rev, N5for and N5rev, respectively. Double mutations (N1+N2, N3+N5, N3+N4 and N4+N5) were created using pSBHACAH1 N1 with primer pair N2for and N2rev, pSBHACAH1 N3 with primer pair N5for and N5rev, and pSBHACAH1 N4 with primer pairs N3for and N3rev or N5for and N5rev, respectively. The triple mutant N3+N4+N5 was created using pSBHACAH1 N4+N5 with primers N3for and N3rev. Quadruple mutants and the non-glycosylated HA-tagged CAH1 (NG) were created by cutting single, double and triple mutants with NcoI and SacI, respectively, and ligating vector backbone and inserts to create Q1 (N2 backbone and N3+N4+N5 insert), Q2 (N1 backbone and N3+N4+N5 insert), Q3 (N1+N2 backbone and N4+N5 insert), Q4 (N1+N2 backbone and N3+N5 insert), Q5 (N1+N2 backbone and N3+N4 insert) and NG (N1+N2 backbone and N3+N4+N5 insert).

Cysteine mutant clones of HA-tagged CAH1 were created by site-directed mutagenesis using PCR with primers that introduce the restriction site corresponding to XhoI. Single mutants were generated by ligating restricted PCR fragments amplified using primers C1 for and C1 rev (C1), C2 for and C2 rev (C2), C3 for and C3 rev (C3) or C4 for and C4 rev (C4), respectively. The C1+C3 double mutant was created by cutting the C1 and C3 mutant clones with NcoI and BsrGI and ligating the C1 vector backbone with the C3 insert fragment.

The DNA sequences of all constructions were verified by DNA sequence analysis and/or restriction analysis to ensure they had the correct sequences and reading frames.

### Establishment of stably transformed *Arabidopsis* cell suspension cultures

To enable *Agrobacterium* mediated plant cell transformation, the N-terminally HA-tagged CAH1 sequence was PCR amplified with ForCAH1/KpnI and RevCAH1/SacI, digested and subcloned into KpnI and SacI digested pMDC32-GFP [41] to create pMDC-HACAH1 containing HACAH1 under the control of a double 35S promoter. pMDC-HACAH1 was transformed into the *Agrobacterium* strain GV3103 (pMP90) and used to transform *Arabidopsis* Ler cell suspension culture.

*Arabidopsis thaliana* cell suspension culture, ecotype *Landsberg erecta (Ler)*, was grown in medium containing Murashige and Skoog basal salt mixture (Sigma, MO 63103, USA) and 1× B5 vitamin mixture [42], supplemented with 3% (w/v) sucrose, 0.5 mg l^−1^ naphtalene acetic acid (NAA, Sigma), 0.05 mg l^−1^ kinetin (Sigma), pH (5.7). *Arabidopsis* cells were maintained by weekly sub-culturing of 3 ml culture into 22 ml fresh medium in 100 ml Erlenmeyer flasks rotated at 120 rpm at 16 h light (22°C) and 8 h dark (18°C) cycle. *Agrobacterium* GV3101 (pMP90) were grown in 2×YT medium. 500 µl of overnight grown culture was pelleted by centrifugation at 5.000 g for 5 min, resuspended in *Arabidopsis* cell suspension medium and added to 12 ml *Arabidopsis* cell suspension culture, 3 days post-inoculation. Cells were co-cultivated for 2 days. *Agrobacterium* was removed by low speed centrifugation at 200 g for 5 min. Plant cells were washed twice in 50 ml 3% (w/v) sucrose and once in 30 ml cell suspension medium. Finally *Arabidopsis* cells were resuspended and cultivated in 25 ml cell suspension medium supplemented with carbenicillin (500 ug/ml) to stop bacterial growth, and hygromycin (20 ug/ml) to select for transformed plant cells. Cells were subcultered approximately every 10 day for 40 days in cell suspension media supplemented with antibiotics. At days 10 and 20, 20 ml cell suspension was added to 30 ml fresh media, at day 30 and 40, 5 ml cell suspension was added to 20 ml fresh media. After 40 days cell suspension was propagated under normal conditions without antibiotics and subcultered by weekly inoculations of 3 ml cell suspension to 22 ml fresh media.

To select for cells showing high expression of HA-tagged CAH1, cells were cultivated on plates containing cell suspension medium supplemented with 0.8% (w/v) agar. After two-three weeks individual calluses were used to start cell suspension cultures. Cell suspension lines were screened by protein extraction and Western blotting using HA-antibodies.

### Immunogold and electron microscopy

Three days after subcultering, *Arabidopsis* cell cultures, wild-type and expressing HACAH1 (HC), were pelleted by centrifugation at 120 g for 7 min and resuspended in 2% (w/v) agar to facilitate blocks being cut. Blocks were fixed in 2.5% (v/v) glutaraldehyde (Fluka, Buchs, Switzerland) in 0.05 M sodium cacodylate solution (pH 7.4). Samples were degasified under vacuum in two periods of 10 min and then incubated for 2.5 h at 4°C, washed with cacodylate buffer and kept at 4°C overnight. Samples were dehydrated in gradual increased ethanol series (30%, 50%, 70%, and 90% respectively), 15 min each, followed by dehydration in 90% (v/v) ethanol in two periods of 30 min and one of 1 h. Fixed samples were embedded in LRWhite resin (London Resin Company, Reading, UK) in 90% (v/v) ethanol (1∶2) overnight, followed by 4 h incubation in LRWhite in 100% (v/v) ethanol (2∶1) at 4°C before pure LRWhite resin was added. Resin change was performed everyday for 3 days. Polymerization was carried out in capsules at 60°C for 48 h. Ultrathin sections (60 nm) of the blocks were mounted in nickel grids for immunogold assays. After blocking in 20 mg/ml BSA in TBS buffer (pH 7.4) for 1 h at 37°C, grids were incubated for 1 h at 37°C with anti-HA primary antibody (1∶500 in blocking solution). Sections were washed five times in TBS buffer containing 2 mg ml-1 BSA and then incubated for 45 min at 37°C in secondary antibody (1∶50 goat anti-mouse IgG-conjugated 10 nm gold particles, Sigma, St. Louis, MO, USA). Grids were rinsed five times in TBS buffer containing 2 mg/mL BSA and three times in TBS buffer containing 0.1% (v/v) Triton X-100 and 2 mg/mL BSA. Last three brief bi-distilled water washings (3 min each) were performed before samples were allowed to dry at room temperature. Grids were post-stained for 5 min with uranyle acetate solution (to saturation in MetOH), washed with distilled water and incubated with Reynolds lead citrate for 45 s under a N_2_ atmosphere to avoid precipitation. After washing, dry sections were analysed in a JEOL JEM 1010 transmission electron microscope (Tokio, Japan), set to 80 KV. Images were captured using a BioScan camera (Gatan, Pleasanton, CA, USA).

### Transient expression in *Arabidopsis* thaliana protoplasts

Plasmid DNA was prepared from *Escherichia coli* DH5-α cells (JETstar Midi, GENOMED) grown overnight, and resuspended in water to about 1 µg µl^−1^.

Protoplasts for transient expression experiments were prepared from *Arabidopsis* suspension culture cells (three days post-inoculation) and transfected using polyethylene glycol (PEG) [43]. Intact protoplasts were prepared by resuspending protoplasts, after centrifugation at 190 *g* for 5 min, in B5 media supplemented with 0.28 M sucrose. Protoplasts floating after 5 min centrifugation at 120 *g* were collected and used for transfection. 50 µl portions of protoplast suspension, each containing 3×10^5^ protoplasts, were transfected with 5 µg plasmid DNA. Transfected protoplasts were washed with 0.275 M Ca(NO_3_)_2_, resuspended in 500 µl B5-0.34 M GM medium [43] and incubated for 20–24 h in darkness at 23°C in 24-well microtiter plate.

*Arabidopsis thaliana* mesophyll protoplasts were isolated from 4 weeks old wt or *cgl1* mutant (C5-5) plants, kindly provided by Dr Antje von Schaewen [24], grown under normal short day conditions. 10 ug of pSBHACAH1 plasmid DNA was transiently transfected by PEG [44] and incubated in W5 medium in microtiter plate as described above.

### Inhibitor treatment of transfected protoplasts

16 h post-transfection, protein biosynthesis was inhibited by addition of cycloheximide (CHX, Sigma) (10 mg/ml in 50% ethanol) to the B5-0.34 M GM medium, final concentration of 100 µM. 8 h after addition of CHX, protoplasts were harvested and extracted protein was treated with or without Endo H and analyzed by SDS-PAGE and Western blotting. N-glycosylation of HC was inhibited by addition of tunicamycin (Sigma, 5 mg/ml in DMSO) to the B5-0.34 M GM medium of the transfected protoplasts, final concentration 20 µg/ml. 20 h post-transfection and tunicamycin addition, protoplasts were harvested and extracted protein was immunoprecipitated using HA-agarose and/or separated by SDS-PAGE and analyzed by Western blotting.

### Preparation of protein extracts,Western blot and affinoblot analysis

10 g of packed *Arabidopsis* cells were frozen in liquid nitrogen and ground with a mortar and pestle. Isolation of soluble and microsome fractions was carried out as previously described [45]. Protoplasts were harvested by two-step centrifugation (7 min at 190 g followed by 30 sec at 5,500 rpm in a microcentrifuge). Proteins in the protoplasts were then prepared for SDS-PAGE by resuspending the resulting pellets in 2× sample buffer [46] under reducing (10% 2-ME, 95°C, 5 min) or non-reducing (no 2-ME, 70°C, 30 min) conditions.

Proteins separated by SDS-PAGE were electro-transferred onto nitrocellulose membrane (Bio-Rad, CA 94547, USA). High mannose-type glycoproteins were detected by affinoblot, as described by Faye and Chrispeels [23] with minor modifications. Briefly, the membrane was blocked in TBS/Tween-20 (TTBS) for one hour, incubated at room temperature in Concanavalin A (Con A, Sigma, USA), buffer (1 mM MnC1_2_, 1 mM CaCl_2_, 0,5 M NaCl, 25 ug ml^−1^ Con A in TBS) for two hours followed by four washing steps, 15 min each, in TTBS. Incubation with 5 ug. ml^−1^ horse radish peroxidase (Sigma, USA) in TTBS for one hour was followed by four washing steps in TTBS and one in TBS, 15 min each, prior to detection.

For immunoblot assays, membranes wereprobed with primary antibodies (α-HA, HA11 from Nordic Biosite AB, Täby, Sweden; antibodies against α(1,3)-fucose residues, AS07 268 from AgriSera, Sweden; or α-BiP, Hsc70 from Nordic Biosite AB) at 1∶1.000 dilution and detected using appropriate horseradish peroxidase-conjugated secondary antibodies (Donkey Anti-Rabbit IgG from GE Healthcare or Goat Anti-Mouse IgG from Bio-Rad) at 1∶10.000 dilution and enhanced chemiluminescence (using an ECL or ECL Advance, Amersham ECL Western Blotting Detection Kit, GE Healthcare, Buckinghamshire, UK). Primary and secondary antibodies were diluted in TBS/Tween-20 supplemented with 2% (w/v) non-fat dry milk and incubated overnight with the membranes at 4°C (for primary antibodies) and one hour at room temperature (secondary antibodies). Membranes were stripped and reprobed with new antibodies according to the recommendations of the ECL kits' manufacturer (GE Healthcare).

Affino and immunoreaction products were detected using a Fujifilm LAS-3000 Luminescent Image Analyzer (Fuji Photo Film Co. Ltd, Tokyo, Japan) operating at standard sensitivity at tray position 1 to ensure high resolution and image quality. Immunoreactions were then quantified using Multi Gauge software (Fuji Photo Film Co. Ltd), and regions of interests (ROIs) were analyzed by subtracting the local background, enabling ROIs of different sizes to be distinguished and compared. To verify that the size of the ROI did not influence our measurements, quantification of test samples were performed. The ROI did not have any significant effect on the estimated signal under our experimental conditions, validating and enabling our further analysis of our samples using this method.

### Deglycosylation using Endo H

Pelleted protoplasts were resuspended in 30 µl extraction buffer – 25 mM Tris-HCl (pH 7.8), 10 mM MgCl_2_, 5 mM EGTA, 2 mM DTT, 10% (v/v) glycerol, 75 mM NaCl, 0.2% (v/v) IgePal-630, 1 mM benzamidine and protease inhibitor cocktail (PIC, Sigma) – and vortex-mixed for 30 s. The protoplasts were then frozen in liquid N_2_, thawed on ice and (after centrifugation in a microcentrifuge) the soluble protein extract was deglycosylated by Endo Hf (New England Biolabs, Hertfordshire, UK). Soluble and microsome fractions from cell suspension cultures were subjected to deglycosylation without further treatment. Briefly, 1 µl denaturing buffer was added to 9 µl of each kind of sample and heated at 100°C for 10 min. The reaction volume was brought to 20 µl by adding 500 U Endo Hf, reaction buffer (New England Biolabs) and PIC then the mixture was incubated overnight at 37°C. Finally, the reaction was stopped and the proteins in each mixture were prepared for SDS-PAGE, as described above.

### Immunoprecipitation of HA-tagged CAH1

40 µl protoplast protein extract was diluted to 250 µl with PBS, then HA-tagged protein was precipitated by incubation with 20 µl (40 µl 1∶1 suspension) anti-HA Agarose Conjugate (Clone HA-7, Sigma) at 4°C overnight using an orbital shaker according to according to Sigma's recommendations. Proteins from *Arabidopsis* suspension culture cells were extracted by grinding in liquid nitrogen and resuspended in RIPA buffer – 50 mM Tris-HCl buffer pH 7.5, 150 mM NaCl, 1% (v/v) NP-40, 0.5% (w/v) DOC (deoxycholic acid, Sigma), 0.1% (w/v) SDS, and PIC (protease inhibitor cocktail, Roche). Protein A/G plus-agarose beads (Santa Cruz, sc-2003) were washed with 10 mM Tris-HCl buffer, pH 8.0, containing 0.1 mM EDTA and 0.02% (w/v) sodium azide, blocked with 2% (w/v) BSA overnight at 4°C and used to pre-clear the protein extract for 2 h at 4°C. A monoclonal anti-HA antibody, obtained by purifying cellular extracts from the fibroblast clone 12c5A, was kindly provided by Dr Martínez Granero (CNIC, Madrid, Spain) and added to the precleared protein extract then the mixture was incubated overnight at 4°C. Following incubation with the antibody, blocked beads were added and the mixture was incubated for a further 2 h at 4°C. Immunoprecipitated material was pelleted by centrifugation at 10,000 g for 5 min and washed once in RIPA buffer. Precipitated (and co-precipitated) protein was prepared for SDS-PAGE or used for Endo H deglycosylation.

### Multiple sequence alignment and 3D-modelling of CAH1

Protein sequence analysis of *Arabidopsis* α-CA homologues was performed using ClustalW2 [47]. 3D structure of CAH1 was predicted using the SWISS-MODEL protein structure homology-modelling server operating in automated mode [48], [49], [50]. Full length amino acid sequence of CAH1 was uploaded and residue 30 to 268 was modelled using 1kopA PDB molecule [51] and visualized using Swiss-PdbViewer [52].

### Membrane-inlet mass spectrometry (MIMS) measurements of CAH1 activity

In order to measure the specific activity of the CAH1 protein and to avoid interference with the activity from the numerous other cellular CA isoforms, measurements were carried out with HA-CAH1 from *Arabidopsis* suspension culture cells stably transformed with tagged CAH1 and immunoprecipitated as described above. As control samples, immunoprecipitated proteins from culture cells not expressing the tagged CAH1 and/or buffer were used. In order to study the importance of N-glycosylation for the activity of CAH1, HA-tagged CAH1 was immunoprecipitated from culture cells treated with tunicamycin for 24 h (10 µg mL^−1^, final concentration). The MIMS measurements of CA activity of all these samples were performed as described earlier [53] by monitoring the change in ^12^C^18^O^18^O concentration (*m/z* = 48) as a function of time after the injection of 15-µl air-saturated H_2_^18^O (97% initial; ≈2.4% final enrichment). An isotope ratio mass spectrometer (*ThermoFinnigan^Plus^ XP*) was connected *via* a cooling trap (dry ice +EtOH) to a home-built membrane-inlet cell similar to that described by Messinger and co-authors [54], but with a larger volume (600 µl). The sample in the cell was separated from the high vacuum (3×10^−8^ bar) of the mass spectrometer *via* a 150 µm-thick metallic mesh silicon membrane (*Franatech GmbH*, Germany) seamlessly resting on a porous Teflon support (*Small Parts Inc.*, USA). The reaction mixture was kept at 20°C and stirred constantly during measurements with a magnetic stir bar. CO_2_ was detected online as the non-labelled (^12^C^16^O^16^O), singly-labelled (^12^C^16^O^18^O), and doubly-labelled (^12^C^18^O^18^O) species at *m/z* = 44, 46 and 48, respectively.

## Supporting Information

Figure S1 **Localization of HA-tagged CAH1 by immunogold labelling and electron microscopy.** (A and B) Transmission Electron Microscopy (TEM) images of wt *Arabidopsis* suspension culture cells. (A) Detailed image of a chloroplast. (B) General view of the cell with ER, Golgi (G), cell wall (CW), vacuole (V), and chloroplast (C). The immunogold (IG) labelling over these sections is barely detectable. (C–E) TEM images of *Arabidopsis* suspension cells stably expressing HC. (C and D) Detailed images of chloroplasts. (E) IG labelling over the endomembrane system and cell wall in these transgenic cells expressing HC. Bar: 0.2 µm.
(TIF)

Figure S2 **HA-tagged CAH1 is glycosylated in plant cells, harbouring four or five N-glycans.** Migration patterns of HA-tagged CAH1 forms in protoplasts of *Arabidopsis* suspension culture cells (A) and *Arabidopsis* mesophyll cells (B) expressing wt (HC) or mutant CAH1. Antibody specificity was verified using water-transfected protoplasts (−). (A) Protoplasts from *Arabidopsis* cell suspension culture transfected with HC and CAH1 variants mutated in 1, 2, 3, 4 and 5 N-glycosylation sites were analyzed using HA-antibodies. Removal of one glycosylation site (N1–N5) resulted in glycoforms migrating faster than wt HC, matching with the removal of one N-glycan. In addition, mutating two (N1+N2, N3+N4, N3+N5 and N4+N5) or three (N3+N4+N5) glycosylation sites resulted in glycoforms that migrated according to the expected weight of HA-tagged CAH1 with three or two remaining N-linked glycans, respectively. Quadruple mutants (Q1–Q5) migrated faster than HA-tagged CAH1 with three glycosylation sites removed (N3+N4+N5), though they still represented glycoforms with a higher molecular mass than the NG mutant. (B) Mutants lacking one of the five potential glycosylation sites (N1–N5) migrate faster than HC protein. (C) Detailed scheme of the migration pattern of HC and single mutant CAH1 forms seen in (B). High mannose type glycoforms are symbolized by squares and complex type glycoforms by triangles. Colours represent glycosylation sites: blue, NAT60; yellow, NYT87; orange, NHT157; gray, NVS194; red, NNS224.
(TIF)

Figure S3 **Enrichment of ER marker in microsome fraction.** Western blot using antibodies against the ER localized protein BiP to verify that microsome fractions in Figure 4a and b were intact. S and I, soluble supernatant and insoluble microsome pellet, respectively.
(TIF)

Figure S4 **CAH1 contains an intramolecular disulphide bridge.** (A) ClustalW2 [47] protein sequence analysis of *Arabidopsis* α-CA homologues. Cysteine residues at positions 27 and 191 (red) in CAH1 sequence are conserved (*) in seven of the eight homologues described by Fabre *et al.* [4]. CA8 is substantially larger than CA1-7, possibly indicating that this gene product is not a functional α-CA or belonging to another group of proteins. (B and C) Proteins from *Arabidopsis* suspension protoplasts expressing HA-tagged wt CAH1 (HC) or C1+C3 double mutant were separated under reducing (+) and non-reducing (−) conditions (with and without 2-mercaptoethanol, 2-ME) and probed with HA antibodies. 2-ME from the reduced sample (+) is diffusing into the middle lane (−), affecting the migration of the non-reduced sample. The C1+C3 double mutant is completely insensitive to reducing agents, since addition of 2-ME to the sample buffer had no effect on the migration pattern of the double mutant, as observed for the HC protein.
(TIF)

Figure S5 **TargetP analysis of** ***Arabidopsis*** **α-type CAs.** TargetP [8] analysis of *Arabidopsis* α-type CAs protein sequences described by Fabre *et al.* [4]. Seven of the eight homologues have an N-terminal signal sequence (SP) for the ER.
(TIF)

Figure S6 **Cycloheximide treatment results in a higher proportion of Endo H resistant HC.** (A) Protoplasts from *Arabidopsis* cell suspension culture were transiently transfected with HA-tagged wt CAH1 (HC). Prior to protein extraction, protoplasts were either incubated in presence or absence of the protein biosynthesis inhibitor cycloheximide (CHX) for 8 h. Extracted proteins were further treated or non-treated with Endo H. Endo H resistant, complex glycoforms, are marked by triangles. Endo H sensitive, high mannose type glycoforms, by squares. (B) Densitometric analysis of the Endo H resistant and sensitive glycoforms of HC from CHX treated and non-treated protoplasts. The graph represents the ratio of resistant/sensitive glycoform (mean ± SE, * = p<0.05, n = 4). Higher ratio corresponds to higher proportion of Endo H resistant, complex type, glycoforms of the protein.
(TIF)

Table S1 **Nomenclature of constructs used to transfect plant cells and protoplasts.**
(DOC)

## References

[1] Khalifah RG (1971). The carbon dioxide hydration activity of carbonic anhydrase. I. Stop-flow kinetic studies on the native human isoenzymes B and C.. J Biol Chem.

[2] Moroney JV, Bartlett SG, Samuelsson G (2001). Carbonic anhydrases in plants and algae.. Plant Cell Environ.

[3] Samuelsson G, Karlsson J, Aro E-M, Andersson B (2001). Chloroplastic Carbonic Anhydrases.. Regulation of Photosynthesis.

[4] Fabre N, Reiter IM, Becuwe-Linka N, Genty B, Rumeau D (2007). Characterization and expression analysis of genes encoding alpha and beta carbonic anhydrases in Arabidopsis.. Plant Cell Environ.

[5] Sawaya MR, Cannon GC, Heinhorst S, Tanaka S, Williams EB (2006). The structure of beta-carbonic anhydrase from the carboxysomal shell reveals a distinct subclass with one active site for the price of two.. J Biol Chem.

[6] Hewett-Emmett D, Tashian RE (1996). Functional diversity, conservation, and convergence in the evolution of the alpha-, beta-, and gamma-carbonic anhydrase gene families.. Mol Phylogenet Evol.

[7] Villarejo A, Buren S, Larsson S, Dejardin A, Monne M (2005). Evidence for a protein transported through the secretory pathway en route to the higher plant chloroplast.. Nat Cell Biol.

[8] Emanuelsson O, Brunak S, von Heijne G, Nielsen H (2007). Locating proteins in the cell using TargetP, SignalP and related tools.. Nat Protoc.

[9] Hebert DN, Molinari M (2007). In and out of the ER: protein folding, quality control, degradation, and related human diseases.. Physiol Rev.

[10] Asatsuma S, Sawada C, Itoh K, Okito M, Kitajima A (2005). Involvement of alpha-amylase I-1 in starch degradation in rice chloroplasts.. Plant Cell Physiol.

[11] Kitajima A, Asatsuma S, Okada H, Hamada Y, Kaneko K (2009). The Rice {alpha}-Amylase Glycoprotein Is Targeted from the Golgi Apparatus through the Secretory Pathway to the Plastids.. Plant Cell.

[12] Chen MH, Liu LF, Chen YR, Wu HK, Yu SM (1994). Expression of alpha-amylases, carbohydrate metabolism, and autophagy in cultured rice cells is coordinately regulated by sugar nutrient.. Plant J.

[13] Chen MH, Huang LF, Li HM, Chen YR, Yu SM (2004). Signal peptide-dependent targeting of a rice alpha-amylase and cargo proteins to plastids and extracellular compartments of plant cells.. Plant Physiol.

[14] Nanjo Y, Oka H, Ikarashi N, Kaneko K, Kitajima A (2006). Rice plastidial N-glycosylated nucleotide pyrophosphatase/phosphodiesterase is transported from the ER-golgi to the chloroplast through the secretory pathway.. Plant Cell.

[15] Jarvis P, Robinson C (2004). Mechanisms of protein import and routing in chloroplasts.. Curr Biol.

[16] Mitra N, Sinha S, Ramya TN, Surolia A (2006). N-linked oligosaccharides as outfitters for glycoprotein folding, form and function.. Trends Biochem Sci.

[17] Parodi AJ (2000). Role of N-oligosaccharide endoplasmic reticulum processing reactions in glycoprotein folding and degradation.. Biochem J.

[18] Maley F, Trimble RB, Tarentino AL, Plummer TH (1989). Characterization of glycoproteins and their associated oligosaccharides through the use of endoglycosidases.. Anal Biochem.

[19] Kornfeld R, Kornfeld S (1985). Assembly of asparagine-linked oligosaccharides.. Annu Rev Biochem.

[20] Bencur P, Steinkellner H, Svoboda B, Mucha J, Strasser R (2005). Arabidopsis thaliana beta1,2-xylosyltransferase: an unusual glycosyltransferase with the potential to act at multiple stages of the plant N-glycosylation pathway.. Biochem J.

[21] Downing WL, Galpin JD, Clemens S, Lauzon SM, Samuels AL (2006). Synthesis of enzymatically active human alpha-L-iduronidase in Arabidopsis cgl (complex glycan-deficient) seeds.. Plant Biotechnol J.

[22] Lerouge P, Cabanes-Macheteau M, Rayon C, Fischette-Laine AC, Gomord V (1998). N-glycoprotein biosynthesis in plants: recent developments and future trends.. Plant Mol Biol.

[23] Faye L, Chrispeels MJ (1985). Characterization of N-linked oligosaccharides by affinoblotting with concanavalin A-peroxidase and treatment of the blots with glycosidases.. Anal Biochem.

[24] von Schaewen A, Sturm A, O'Neill J, Chrispeels MJ (1993). Isolation of a mutant Arabidopsis plant that lacks N-acetyl glucosaminyl transferase I and is unable to synthesize Golgi-modified complex N-linked glycans.. Plant Physiol.

[25] Shental-Bechor D, Levy Y (2008). Effect of glycosylation on protein folding: a close look at thermodynamic stabilization.. Proc Natl Acad Sci U S A.

[26] Wujek P, Kida E, Walus M, Wisniewski KE, Golabek AA (2004). N-glycosylation is crucial for folding, trafficking, and stability of human tripeptidyl-peptidase I.. J Biol Chem.

[27] Williams DB (2006). Beyond lectins: the calnexin/calreticulin chaperone system of the endoplasmic reticulum.. J Cell Sci.

[28] Li J, Zhao-Hui C, Batoux M, Nekrasov V, Roux M (2009). Specific ER quality control components required for biogenesis of the plant innate immune receptor EFR.. Proc Natl Acad Sci U S A.

[29] Hong Z, Jin H, Tzfira T, Li J (2008). Multiple mechanism-mediated retention of a defective brassinosteroid receptor in the endoplasmic reticulum of Arabidopsis.. Plant Cell.

[30] Sung DY, Vierling E, Guy CL (2001). Comprehensive expression profile analysis of the Arabidopsis Hsp70 gene family.. Plant Physiol.

[31] Crofts AJ, Leborgne-Castel N, Pesca M, Vitale A, Denecke J (1998). BiP and calreticulin form an abundant complex that is independent of endoplasmic reticulum stress.. Plant Cell.

[32] Kjaer S, Ibanez CF (2003). Intrinsic susceptibility to misfolding of a hot-spot for Hirschsprung disease mutations in the ectodomain of RET.. Hum Mol Genet.

[33] Forsayeth JR, Gu Y, Hall ZW (1992). BiP forms stable complexes with unassembled subunits of the acetylcholine receptor in transfected COS cells and in C2 muscle cells.. J Cell Biol.

[34] Waheed A, Okuyama T, Heyduk T, Sly WS (1996). Carbonic anhydrase IV: purification of a secretory form of the recombinant human enzyme and identification of the positions and importance of its disulfide bonds.. Arch Biochem Biophys.

[35] Husic HD (1991). Extracellular carbonic anhydrase of Chlamydomonas reinhardtii: localization, structural properties, and catalytic properties.. Canadian Journal of Botany.

[36] Hilvo M, Tolvanen M, Clark A, Shen B, Shah GN (2005). Characterization of CA XV, a new GPI-anchored form of carbonic anhydrase.. Biochem J.

[37] Price GD, Caemmerer S, Evans YR, Yu Y-W, Lloyd Y (1994). Specific reduction of chloroplast carbonic anhydrase activity by antisense RNA in transgenic tobacco plants has a minor effect on photosynthetic CO2 assimilation.. Planta.

[38] Badger MR, Price GD (1994). THE ROLE OF CARBONIC ANHYDRASE IN PHOTOSYNTHESIS.. Annu Rev Plant Physiol Plant Mol Biol.

[39] Jin H, Yan Z, Nam KH, Li J (2007). Allele-specific suppression of a defective brassinosteroid receptor reveals a physiological role of UGGT in ER quality control.. Mol Cell.

[40] Hancock KR, Phillips LD, White DW, Ealing PM (1997). pPE1000: a versatile vector for the expression of epitope-tagged foreign proteins in transgenic plants.. Biotechniques.

[41] Curtis MD, Grossniklaus U (2003). A gateway cloning vector set for high-throughput functional analysis of genes in planta.. Plant Physiol.

[42] Song CJ, Steinebrunner I, Wang XZ, Stout SC, Roux SJ (2006). Extracellular ATP induces the accumulation of superoxide via NADPH oxidases in Arabidopsis.. Plant Physiology.

[43] Leung J, Orfanidi S, Chefdor F, Meszaros T, Bolte S (2006). Antagonistic interaction between MAP kinase and protein phosphatase 2C in stress recovery.. Plant Science.

[44] Yoo SD, Cho YH, Sheen J (2007). Arabidopsis mesophyll protoplasts: a versatile cell system for transient gene expression analysis.. Nat Protoc.

[45] Gasparian M, Pusterla M, Baldan B, Downey PM, Rossetto O (2000). Identification and characterization of an 18-kilodalton, VAMP-like protein in suspension-cultured carrot cells.. Plant Physiol.

[46] Richardson MC, Ingamells S, Simonis CD, Cameron IT, Sreekumar R (2009). Stimulation of lactate production in human granulosa cells by metformin and potential involvement of adenosine 5′ monophosphate-activated protein kinase.. J Clin Endocrinol Metab.

[47] Larkin MA, Blackshields G, Brown NP, Chenna R, McGettigan PA (2007). Clustal W and Clustal X version 2.0.. Bioinformatics.

[48] Arnold K, Bordoli L, Kopp J, Schwede T (2006). The SWISS-MODEL workspace: a web-based environment for protein structure homology modelling.. Bioinformatics.

[49] Kiefer F, Arnold K, Kunzli M, Bordoli L, Schwede T (2009). The SWISS-MODEL Repository and associated resources.. Nucleic Acids Res.

[50] Peitsch MC (1995). Protein modelling by e-mail.. Bio/Technology.

[51] Huang S, Xue Y, Sauer-Eriksson E, Chirica L, Lindskog S (1998). Crystal structure of carbonic anhydrase from Neisseria gonorrhoeae and its complex with the inhibitor acetazolamide.. J Mol Biol.

[52] Guex N, Peitsch MC (1997). SWISS-MODEL and the Swiss-PdbViewer: an environment for comparative protein modeling.. Electrophoresis.

[53] Clausen J, Beckmann K, Junge W, Messinger J (2005). Evidence that bicarbonate is not the substrate in photosynthetic oxygen evolution.. Plant Physiol.

[54] Messinger J, Badger M, Wydrzynski T (1995). Detection of one slowly exchanging substrate water molecule in the S3 state of photosystem II.. Proc Natl Acad Sci U S A.

