# Supplementary figures and images for: Importance of Post-Translational Modifications for Functionality of a Chloroplast-Localized Carbonic Anhydrase (CAH1) in Arabidopsis thaliana
Source: PLoS One. 2011 Jun 10;6(6):e21021. doi: 10.1371/journal.pone.0021021 (PMC3112209; doi:10.1371/journal.pone.0021021)

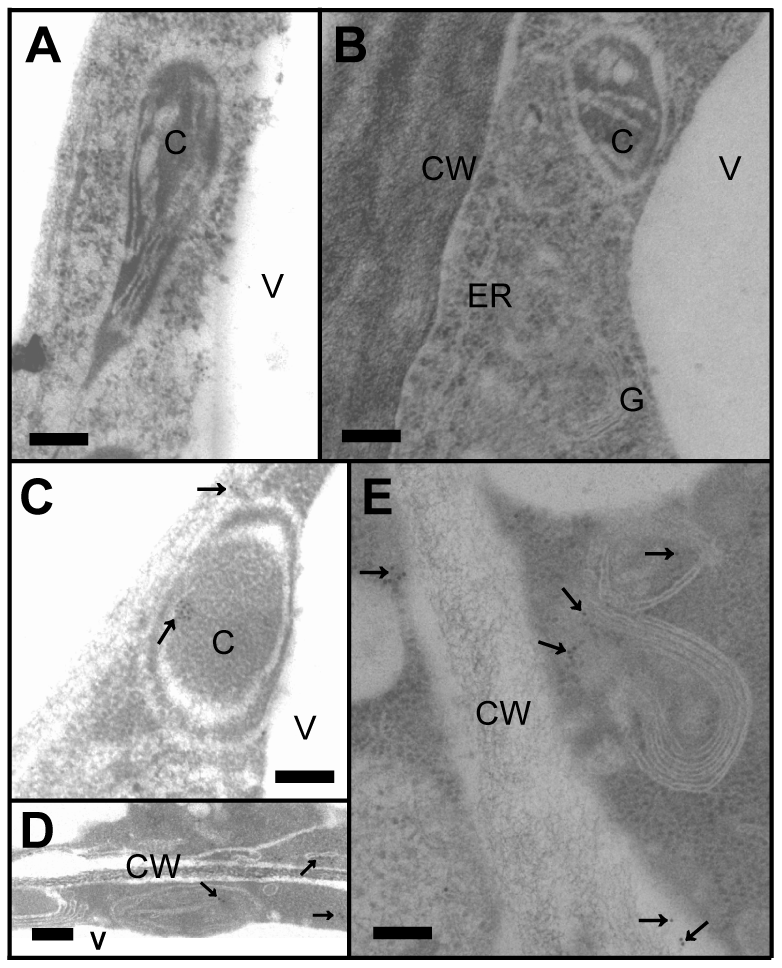

Supplement: Figure S1 — Localization of HA-tagged CAH1 by immunogold labelling and electron microscopy. (A and B) Transmission Electron Microscopy (TEM) images of wt Arabidopsis suspension culture cells. (A) Detailed image of a chloroplast. (B) General view of the cell with ER, Golgi (G), cell wall (CW), vacuole (V), and chloroplast (C). The immunogold (IG) labelling over these sections is barely detectable. (C–E) TEM images of Arabidopsis suspension cells stably expressing HC. (C and D) Detailed images of chloroplasts. (E) IG labelling over the endomembrane system and cell wall in these transgenic cells expressing HC. Bar: 0.2 µm. (TIF) [file pone.0021021.s001.tif]

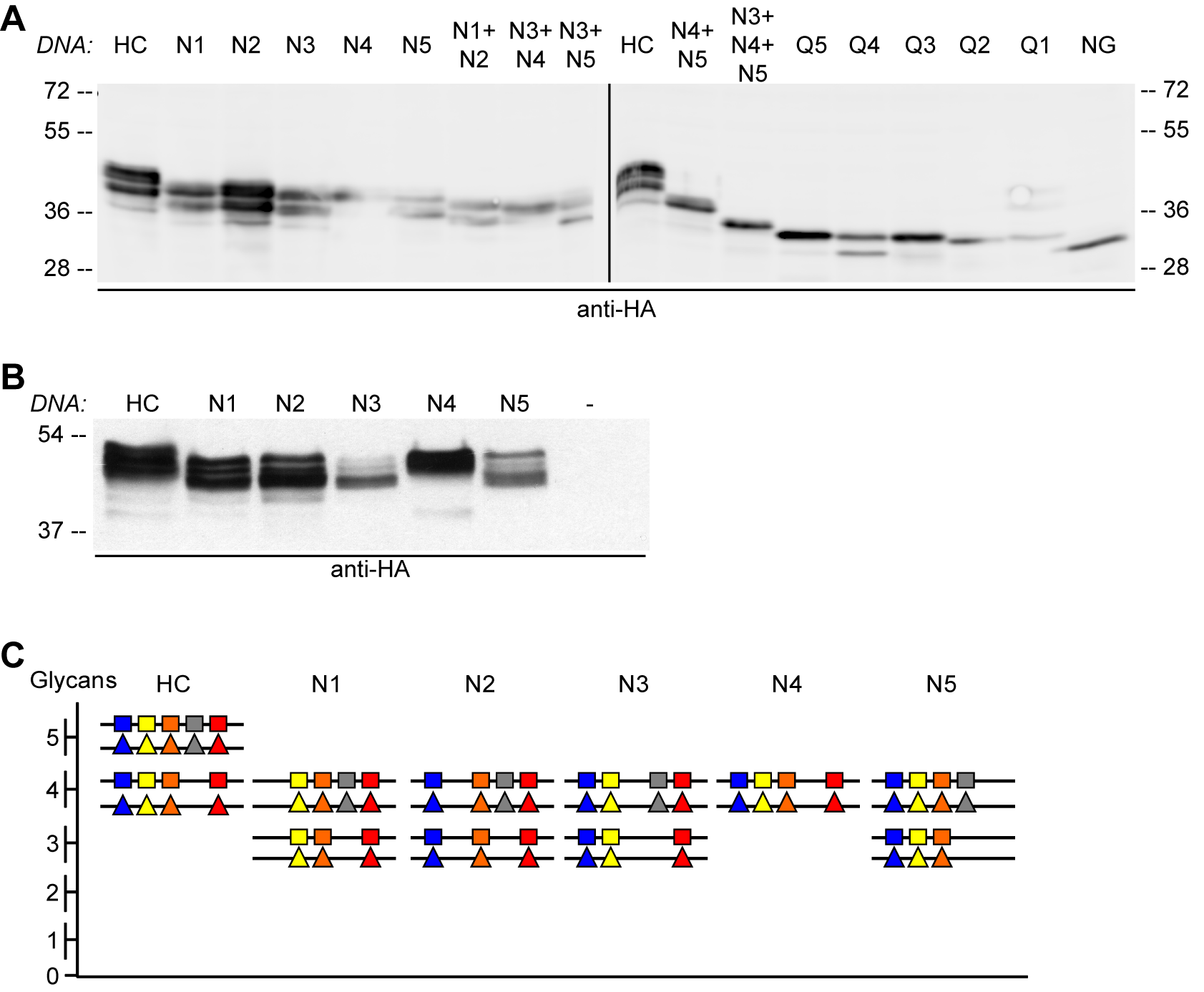

Supplement: Figure S2 — HA-tagged CAH1 is glycosylated in plant cells, harbouring four or five N-glycans. Migration patterns of HA-tagged CAH1 forms in protoplasts of Arabidopsis suspension culture cells (A) and Arabidopsis mesophyll cells (B) expressing wt (HC) or mutant CAH1. Antibody specificity was verified using water-transfected protoplasts (−). (A) Protoplasts from Arabidopsis cell suspension culture transfected with HC and CAH1 variants mutated in 1, 2, 3, 4 and 5 N-glycosylation sites were analyzed using HA-antibodies. Removal of one glycosylation site (N1–N5) resulted in glycoforms migrating faster than wt HC, matching with the removal of one N-glycan. In addition, mutating two (N1+N2, N3+N4, N3+N5 and N4+N5) or three (N3+N4+N5) glycosylation sites resulted in glycoforms that migrated according to the expected weight of HA-tagged CAH1 with three or two remaining N-linked glycans, respectively. Quadruple mutants (Q1–Q5) migrated faster than HA-tagged CAH1 with three glycosylation sites removed (N3+N4+N5), though they still represented glycoforms with a higher molecular mass than the NG mutant. (B) Mutants lacking one of the five potential glycosylation sites (N1–N5) migrate faster than HC protein. (C) Detailed scheme of the migration pattern of HC and single mutant CAH1 forms seen in (B). High mannose type glycoforms are symbolized by squares and complex type glycoforms by triangles. Colours represent glycosylation sites: blue, NAT60; yellow, NYT87; orange, NHT157; gray, NVS194; red, NNS224. (TIF) [file pone.0021021.s002.tif]

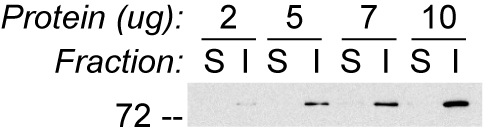

Supplement: Figure S3 — Enrichment of ER marker in microsome fraction. Western blot using antibodies against the ER localized protein BiP to verify that microsome fractions in Figure 4a and b were intact. S and I, soluble supernatant and insoluble microsome pellet, respectively. (TIF) [file pone.0021021.s003.tif]

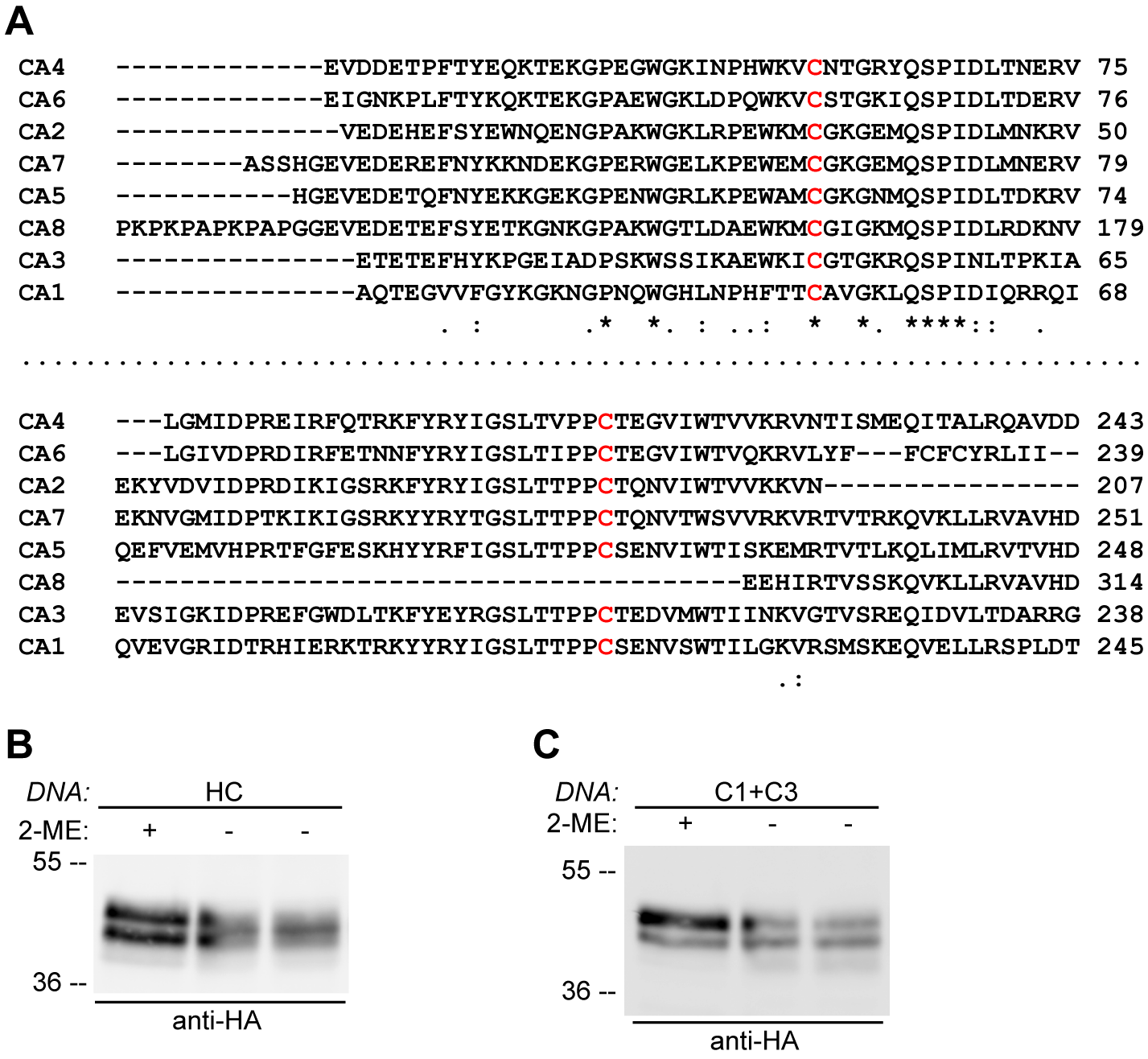

Supplement: Figure S4 — CAH1 contains an intramolecular disulphide bridge. (A) ClustalW2 [47] protein sequence analysis of Arabidopsis α-CA homologues. Cysteine residues at positions 27 and 191 (red) in CAH1 sequence are conserved (*) in seven of the eight homologues described by Fabre et al. [4]. CA8 is substantially larger than CA1-7, possibly indicating that this gene product is not a functional α-CA or belonging to another group of proteins. (B and C) Proteins from Arabidopsis suspension protoplasts expressing HA-tagged wt CAH1 (HC) or C1+C3 double mutant were separated under reducing (+) and non-reducing (−) conditions (with and without 2-mercaptoethanol, 2-ME) and probed with HA antibodies. 2-ME from the reduced sample (+) is diffusing into the middle lane (−), affecting the migration of the non-reduced sample. The C1+C3 double mutant is completely insensitive to reducing agents, since addition of 2-ME to the sample buffer had no effect on the migration pattern of the double mutant, as observed for the HC protein. (TIF) [file pone.0021021.s004.tif]

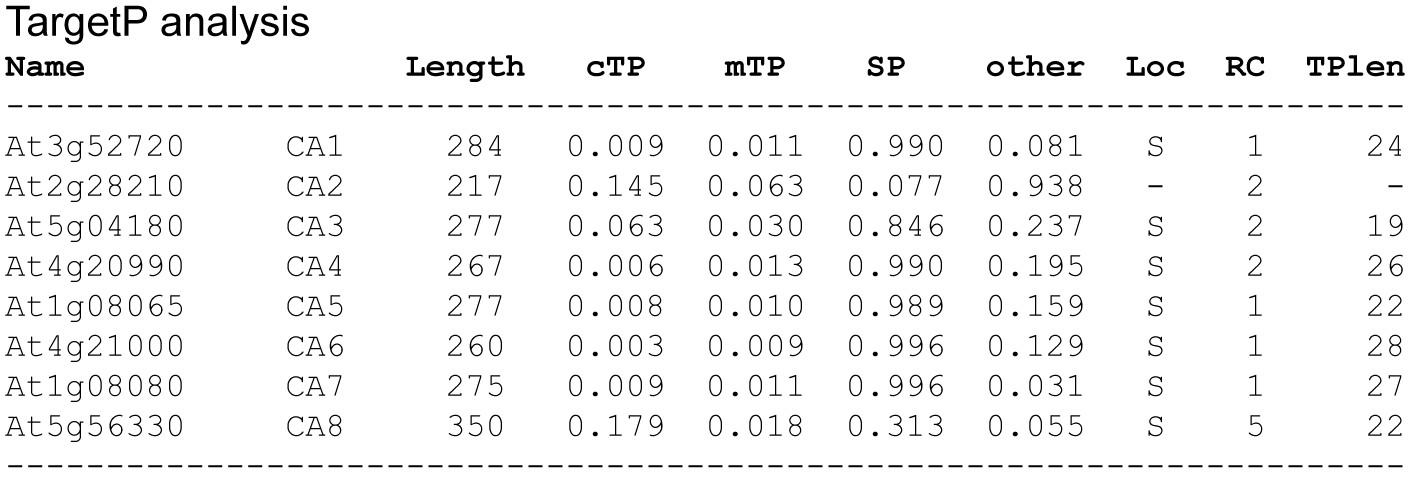

Supplement: Figure S5 — TargetP analysis of Arabidopsis α-type CAs. TargetP [8] analysis of Arabidopsis α-type CAs protein sequences described by Fabre et al. [4]. Seven of the eight homologues have an N-terminal signal sequence (SP) for the ER. (TIF) [file pone.0021021.s005.tif]

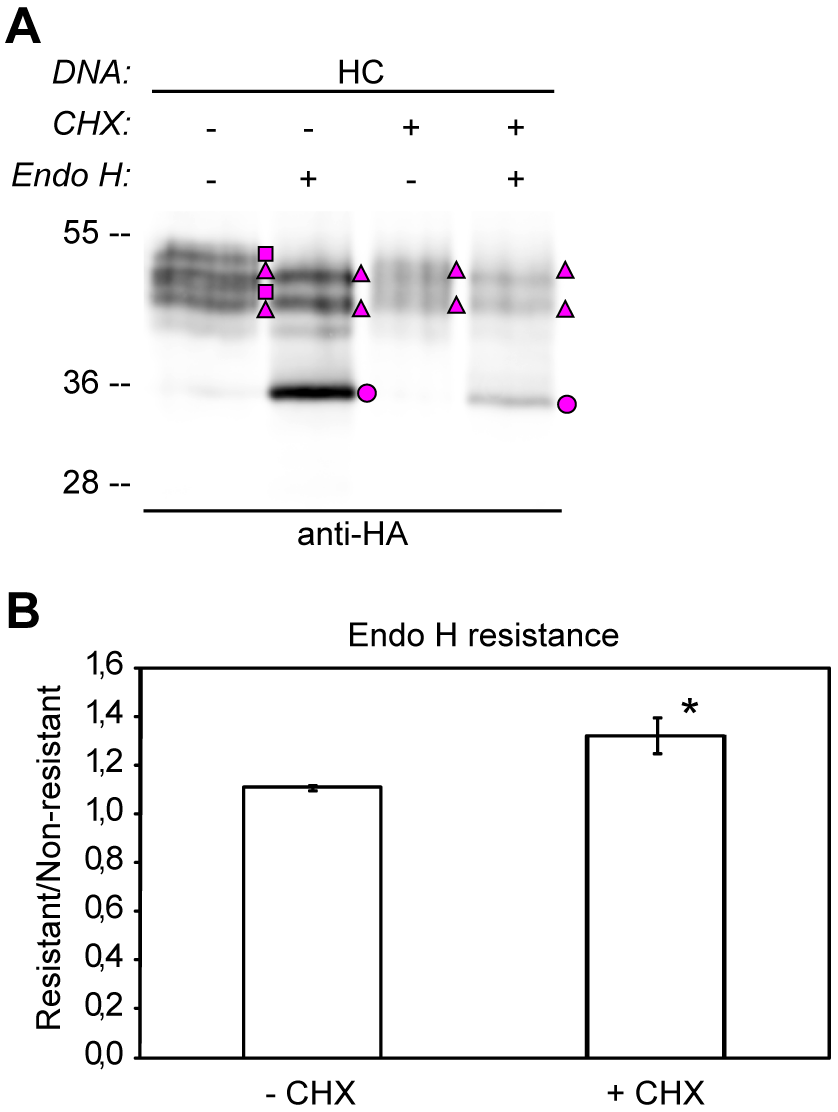

Supplement: Figure S6 — Cycloheximide treatment results in a higher proportion of Endo H resistant HC. (A) Protoplasts from Arabidopsis cell suspension culture were transiently transfected with HA-tagged wt CAH1 (HC). Prior to protein extraction, protoplasts were either incubated in presence or absence of the protein biosynthesis inhibitor cycloheximide (CHX) for 8 h. Extracted proteins were further treated or non-treated with Endo H. Endo H resistant, complex glycoforms, are marked by triangles. Endo H sensitive, high mannose type glycoforms, by squares. (B) Densitometric analysis of the Endo H resistant and sensitive glycoforms of HC from CHX treated and non-treated protoplasts. The graph represents the ratio of resistant/sensitive glycoform (mean ± SE, * = p<0.05, n = 4). Higher ratio corresponds to higher proportion of Endo H resistant, complex type, glycoforms of the protein. (TIF) [file pone.0021021.s006.tif]
