# Supplementary material for: Importance of Post-Translational Modifications for Functionality of a Chloroplast-Localized Carbonic Anhydrase (CAH1) in Arabidopsis thaliana
Source: PLoS One. 2011 Jun 10;6(6):e21021. doi: 10.1371/journal.pone.0021021 (PMC3112209; doi:10.1371/journal.pone.0021021)
Supplement: Table S1 — Nomenclature of constructs used to transfect plant cells and protoplasts. (DOC) [file pone.0021021.s007.doc]

| **Clone** | **Mutated sites** |
| --- | --- |
| N1+N2 | Glycosylation site 1 and 2 |
| N3+N5 | Glycosylation site 3 and 5 |
| N3+N4 | Glycosylation site 3 and 4 |
| N4+N5 | Glycosylation site 4 and 5 |
| N3+N4+N5 | Glycosylation site 3, 4 and 5 |
